# Causal Associations of Epigenetic Age Acceleration With Stroke and Its Functional Outcome: A Two‐Sample, Two‐Step Mendelian Randomization Study

**DOI:** 10.1002/brb3.70412

**Published:** 2025-03-18

**Authors:** Baizhi Qiu, Shuyang Wen, Zifan Li, Yuxin Cai, Qi Zhang, Yuting Zeng, Shuqi Zheng, Zhishan Lin, Yupeng Xiao, Jihua Zou, Guozhi Huang, Qing Zeng

**Affiliations:** ^1^ Department of Rehabilitation Medicine Zhujiang Hospital, Southern Medical University Guangzhou China; ^2^ School of Nursing Southern Medical University Guangzhou China; ^3^ School of Rehabilitation Medicine Southern Medical University Guangzhou China; ^4^ Faculty of Health and Social Sciences The Hong Kong Polytechnic University Hong Kong China

**Keywords:** epigenetic age, mediation, Mendelian randomization, stroke

## Abstract

**Background:**

Emerging evidence from observational studies suggested that epigenetic age acceleration may result in an increased incidence of stroke and poorer functional outcomes after a stroke. However, the causality of these associations remains controversial and may be confounded by bias. We aimed to investigate the causal effects of epigenetic age on stroke and its functional outcomes.

**Methods:**

We conducted a two‐sample Mendelian randomization (MR) analysis to explore the causal relationships between epigenetic age and stroke and its outcomes. Additionally, a two‐step MR analysis was performed to investigate whether lifestyle factors affect stroke via epigenetic age. Datasets of epigenetic age were obtained from a recent meta‐analysis (*n* = 34,710), while those of stroke and its outcomes were sourced from the MEGASTROKE (*n* = 520,000) consortium and Genetics of Ischaemic Stroke Functional Outcome (GISCOME) network (*n* = 6165).

**Results:**

Two‐sample MR analysis revealed a causal relationship between PhenoAge and small vessel stroke (SVS) (OR = 1.07; 95% CI, 1.03–1.12; *p* = 2.01 × 10^−3^). Mediation analysis through two‐step MR indicated that the increased risk of SVS due to smoking initiation was partially mediated by PhenoAge, with a mediation proportion of 9.5% (95% CI, 1.6%–20.6%). No causal relationships were identified between epigenetic age and stroke outcomes.

**Conclusions:**

Our study supports using epigenetic age as a biomarker to predict stroke occurrence. Interventions specifically aimed at decelerating epigenetic aging, such as specific lifestyle changes, offer effective strategies for reducing stroke risk.

## Graphical abstract

Assumption and design for the MR study: (1) two‐sample Mendelian randomized (MR) study and (2) two‐step mediation MR study. Our study supports using epigenetic age as a biomarker to predict stroke occurrence. Changes in specific lifestyle factors may ultimately reduce the risk of stroke by slowing down epigenetic age.

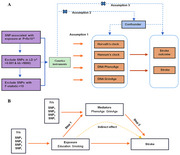

## 1 Introduction

Stroke and its ensuing consequences are the leading causes of global disability and mortality, substantially impacting patients’ quality of life (Saini et al. 2021). Various factors, including age, infarct size, location, heredity, and the extent of brain regeneration (Ospel et al. 2021; Bouslama et al. 2022), influence the neurological outcomes after a stroke.

Epigenetics has emerged as a crucial factor in stroke pathogenesis and recovery, regulating tissue repair through the modulation of gene expression (L. Zhang et al. 2020). Unlike DNA sequence modifications, epigenetic changes result from interactions between the environment and the genome, affecting gene expression without changing the underlying DNA sequence (Horvath 2013). Epigenetic age, as assessed through DNA methylation (DNAm) and molecular markers, provides insights into an individual's health status and rate of agingEpigenetic clocks (Liu et al. 2020), derived from DNAm data, serve as heritable markers of biological aging based on specific sets of CpG sites (Hannum et al. 2013). HannumAge relies on 71 age‐related CpGs in blood, whereas Intrinsic HorvathAge uses 353 age‐related CpGs obtained from different tissues, adjusted for blood cell counts. PhenoAge and GrimAge can help to predict age‐related mortality (Levine et al. 2018). PhenoAge utilizes data from 513 CpGs linked to mortality and clinical biomarkers (Lu et al. 2019), whereas GrimAge integrates data from 1030 CpGs and plasma proteins. Increasing evidence indicates that epigenetic age is involved in stroke pathology, with specific DNAm patterns even predicting stroke occurrence as potential therapeutic targets (Cullell et al. 2022). Additionally, epigenetic age, estimated via DNAm, is believed to predict 3‐month mortality after ischemic stroke. Given that stroke is one of the primary causes of disability and mortality, developing a comprehensive understanding of its pathogenesis beyond traditional risk factors is essential (Qureshi and Mehler 2010; Dichgans et al. 2019).

As the causal relationships between epigenetic age and stroke and its functional outcomes remain unclear, our study aimed to investigate these relationships. We also investigated the effects of lifestyle factors, such as smoking and education. Mendelian randomization (MR), which employs genetic variation as an instrument, provides insights into the causal relationship between exposures and outcomes. A meta‐analysis of genome‐wide association studies (GWAS) conducted in 2021 identified 137 genetic loci associated with epigenetic age acceleration, thus forming the foundation for MR analysis (Smith and Ebrahim 2003). Compared to traditional observational methods, MR is less likely to be affected by confounders and reverse causation, making it a valuable tool for demonstrating causal associations.

## 2 Methods

### 2.1 Study Design

**FIGURE 1:**
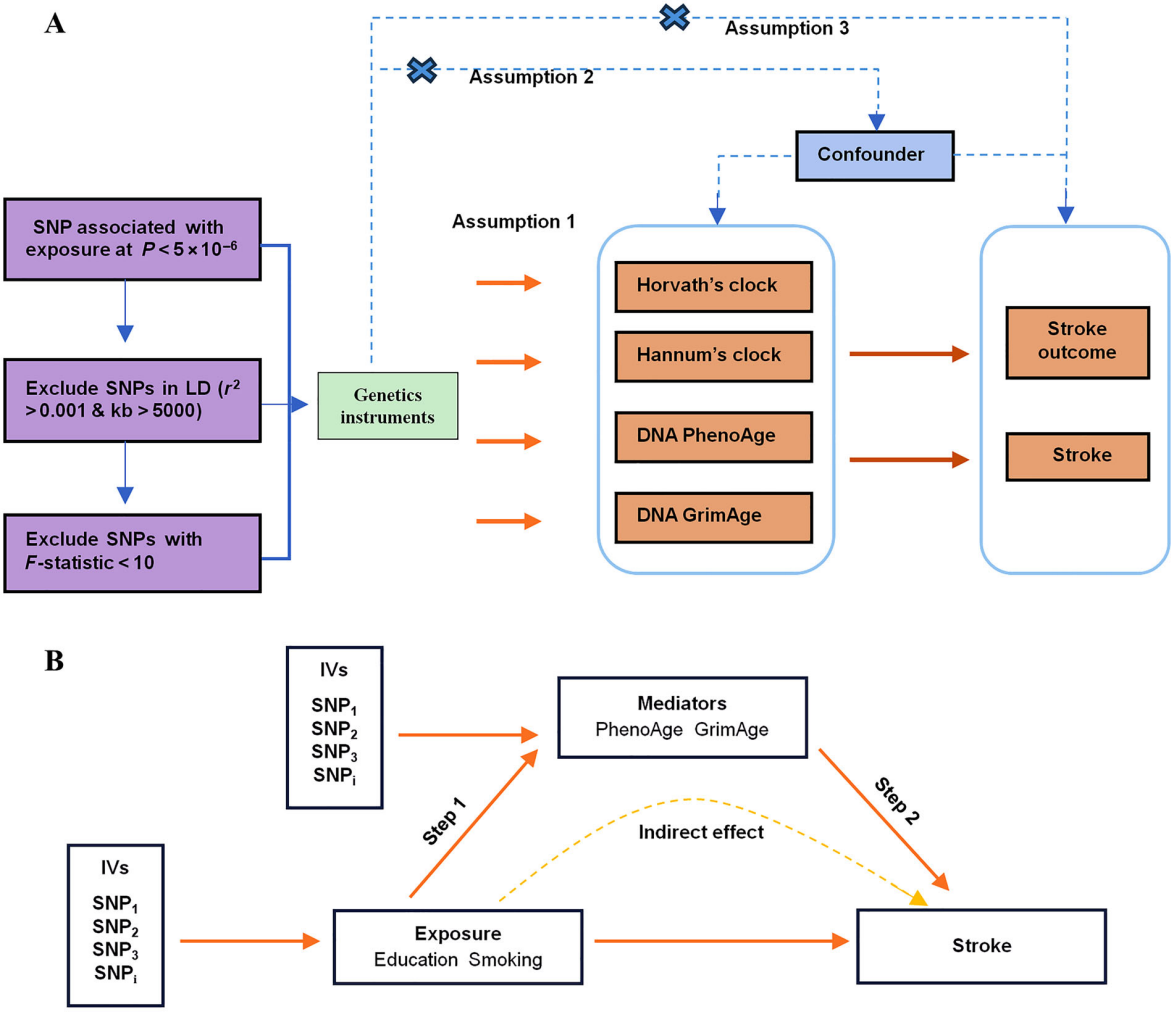
Assumption and design for the MR study. Firstly, a two‐sample MR was performed to investigate the causal relationships between epigenetic age acceleration, stroke, and functional outcome. Secondly, educational attainment and smoking initiation were selected for subsequent two‐step MR analysis (Step 1, the effect of educational attainment and smoking initiation on stroke; Step 2, the effect of epigenetic age acceleration on stroke).

We used a two‐sample univariable MR design to explore genetically determined causal effects between epigenetic age acceleration and stroke and its functional outcomes. Epigenetic age acceleration served as the exposure, measured using various epigenetic clocks (GrimAge clock, PhenoAge clock, Intrinsic Epigenetic Age Acceleration, and HannumAge clock), to investigate potential causal effects on stroke and its functional outcomes. Additionally, a two‐step MR analysis was performed to explore potential mediators and their mediator ratios. Three shared common lifestyle factors were selected from previous studies on epigenetic age and stroke to investigate whether they affect stroke and its functional outcomes through epigenetic age acceleration. To obtain unbiased estimates of the causal effects, the MR analysis was required to satisfy three basic assumptions: (1) the genetic instrumental variables (IVs) should be strongly associated with the exposure, (2) they should not be associated with any potential confounding factors, and (3) they should influence the outcomes solely through the exposure, without any direct or indirect routes. Figure 1 provides an overview of our study design.

### 2.2 Data Sources and Selection Rationale

#### 2.2.1 Epigenetic Age Acceleration

Genetic instruments for epigenetic age acceleration were selected from a large‐scale meta‐analysis of GWAS involving 34,710 individuals of European ancestry from 28 cohorts (Mccartney et al. 2021). The original GWAS included Hannum's clock, Horvath's clock, DNAm PhenoAge, and DNAm GrimAge as measures of epigenetic age acceleration, all expressed as biological aging rates in years. Single‐nucleotide polymorphisms (SNPs) associated with epigenetic age acceleration at genome‐wide significance (*p* < 5 × 10^−8^) were selected as IVs after clumping summary statistics for a linkage disequilibrium (LD) threshold of *r* ^2^ < 0.001 and a distance of > 5000 kb. Since there were few SNPs selected for epigenetic age acceleration, we set a loose threshold (*p* < 5 × 10^−6^) to obtain a relatively appropriate number of IVs.

#### 2.2.2 Lifestyle Factors

**FIGURE 2:**
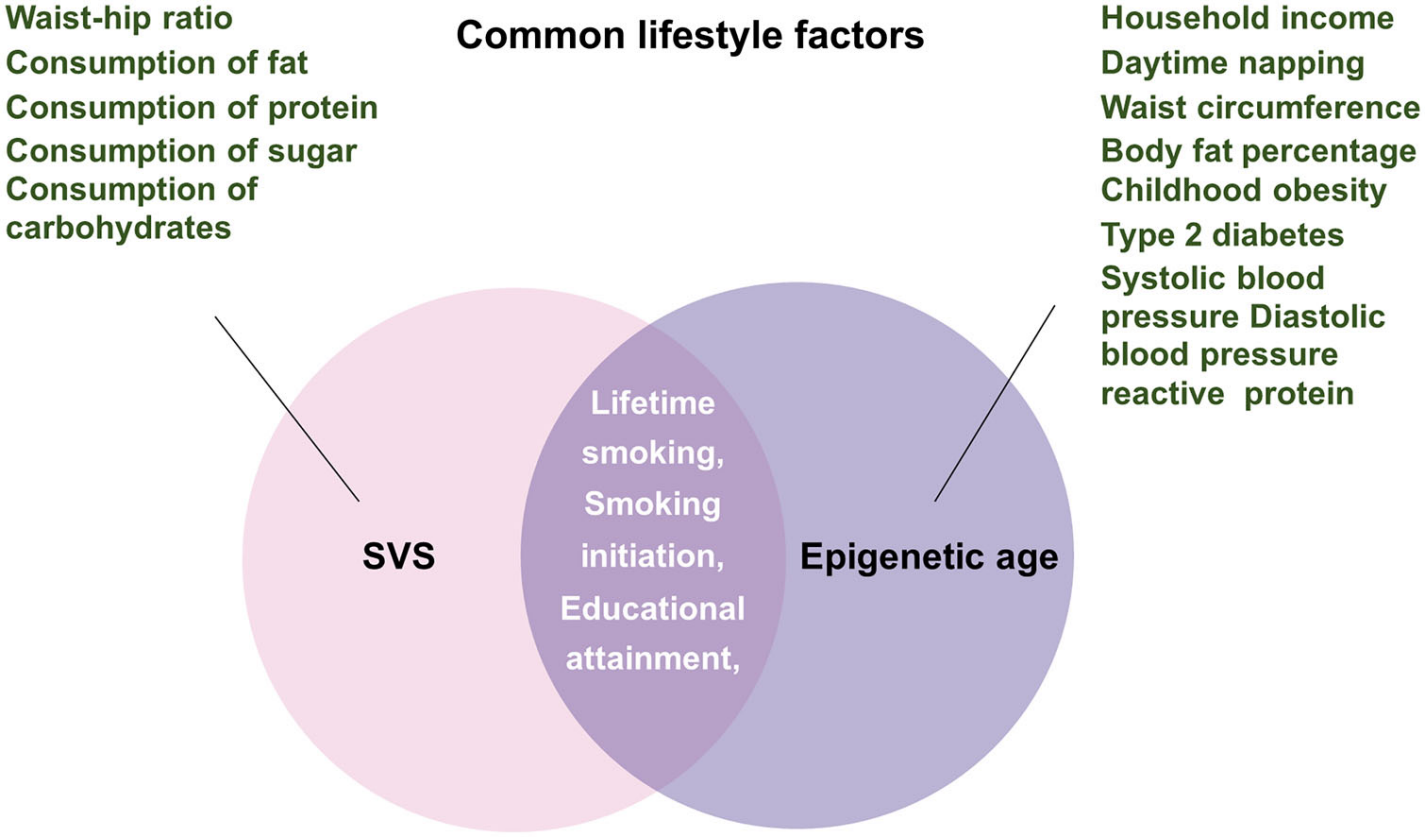
The common lifestyle factors between SVS and epigenetic age. The merged results were selected for subsequent two‐step MR analysis.

Additionally, a two‐step MR analysis was performed to identify potential mediators. These factors are depicted in Figure 2. To ensure the independence of the SNPs from lifestyle factors, IVs were selected at genome‐wide significance (*p* < 5 × 10^−8^), with an LD threshold of *r* ^2^ < 0.001 and a distance of > 10,000 kb.

#### 2.2.3 Stroke and Its Functional Outcomes

The outcome data for stroke and its subtypes were obtained from a recent large GWAS meta‐analysis conducted by the MEGASTROKE consortium, which included data on any stroke (AS), any ischemic stroke (AIS), large‐artery stroke (LAS), SVS, and cardioembolic stroke (CES). Summary statistical data for the functional outcomes of ischemic stroke were retrieved from a GWAS meta‐analysis conducted by the GISCOME network, which included 12 European cohorts (Söderholm et al. 2019). The functional outcomes were assessed using the modified Rankin Scale, with scores ranging 0–6 (where 0 represents *no symptoms* and 6 represents *death*), approximately 3 months after an ischemic stroke. Lower scores (0–2) indicate *better outcomes*, whereas higher scores (3–6) indicate *poorer outcomes*. The scale consisted of two binary variables (0–2 vs. 3–6 and 0–1 vs. 2–6) and a continuous variable. The GWAS results were adjusted for age, sex, and baseline National Institutes of Health Stroke Scale scores in one set and for age and sex only in another set.

### 2.3 Statistical Analysis

#### 2.3.1 Two‐Sample MR Analysis

We performed comprehensive screening using a random‐effects inverse variance weighted (IVW) approach to investigate the relationship between epigenetic age acceleration and stroke, including its functional outcomes (Burgess et al. 2013). Additional methods, such as MR‐Egger and weighted median, were applied to verify the potential associations. To account for multiple testing in the main IVW results, we used the false discovery rate (FDR) method to eliminate false positives.

#### 2.3.2 Two‐Step MR Analysis

We conducted a two‐step MR analysis to determine whether lifestyle factors could influence stroke through epigenetic age acceleration. In the first step, we examined whether these lifestyle factors influenced SVS after potential confounders were excluded (such as BMI, pressure, obesity, and so on). Only lifestyle factors with positive associations were considered for further two‐step MR analysis. In the first step of the two‐step MR, we explored the causal effects of lifestyle factors on epigenetic age acceleration. In the second step, we examined the causal effects of epigenetic age acceleration on stroke. To ensure the validity of the mediation model, we conducted MR Steiger tests to assess the directionality between the mediator, exposures, and outcomes. The mediation proportion was calculated using the total effect and indirect effect, with 95% confidence intervals (CIs) computed using the Delta method (Carter et al. 2021). Additionally, we used the Sobel test to validate the mediation effect (Baron and Kenny 1986).

### 2.4 MR Sensitivity Analysis

To estimate the robustness and reliability of our results, we used Cochran's *Q*‐test to measure heterogeneity and MR‐Egger regression's nonzero intercept to assess horizontal pleiotropy (Bowden et al. 2015, 2016, 2015, 2016). Furthermore, we used the MR‐Pleiotropy Residual Sum and Outlier (MR‐PRESSO) global test to identify and exclude any horizontal pleiotropic outliers (*p* < 0.05) (Verbanck et al. 2018). To prevent weak instruments from influencing our analysis, we calculated *F*‐statistics and the $R^{2}$ for each SNP selected for epigenetic age acceleration (Papadimitriou et al. 2020). The following formulas were used to calculate the strength of each SNP and $R^{2}$:

$$F=R^{2}\left(N-2\right)/\left(1-R^{2}\right)$$

$$\begin{array}{ccc} R^{2} & = & \left[2\times EAF\times \left(1-EAF\right)\times beta^{2}\right]/\\ & & \left[\left(2\times EAF\times \left(1-EAF\right)\times beta^{2}\right)\right.\\ & & \left.+\left(2\times EAF\times \left(1-EAF\right)\times N\times SE{\left(beta\right)}^{2}\right)\right], \end{array}$$

where $R^{2}$ represents the proportion of variation in exposure explained by the SNPs, *N* is the sample size, EAF is the effect allele frequency, beta is the estimated genetic effect, and SE (beta) is the standard error of the genetic effect (Papadimitriou et al. 2020). Finally, to investigate whether the causal effects were primarily influenced by a single SNP, we used a leave‐one‐out approach. All selected genetic instruments were examined in Phenoscanner to ensure that each genetic instrument was associated only with the exposure.

MR analyses were performed using R Studio (version 4.3.1) with various R packages, including TwoSample MR, MR‐PRESSO, RMediation, and Phenoscanner.

## 3 Results

### 3.1 Two‐Sample MR Analysis

**FIGURE 3:**
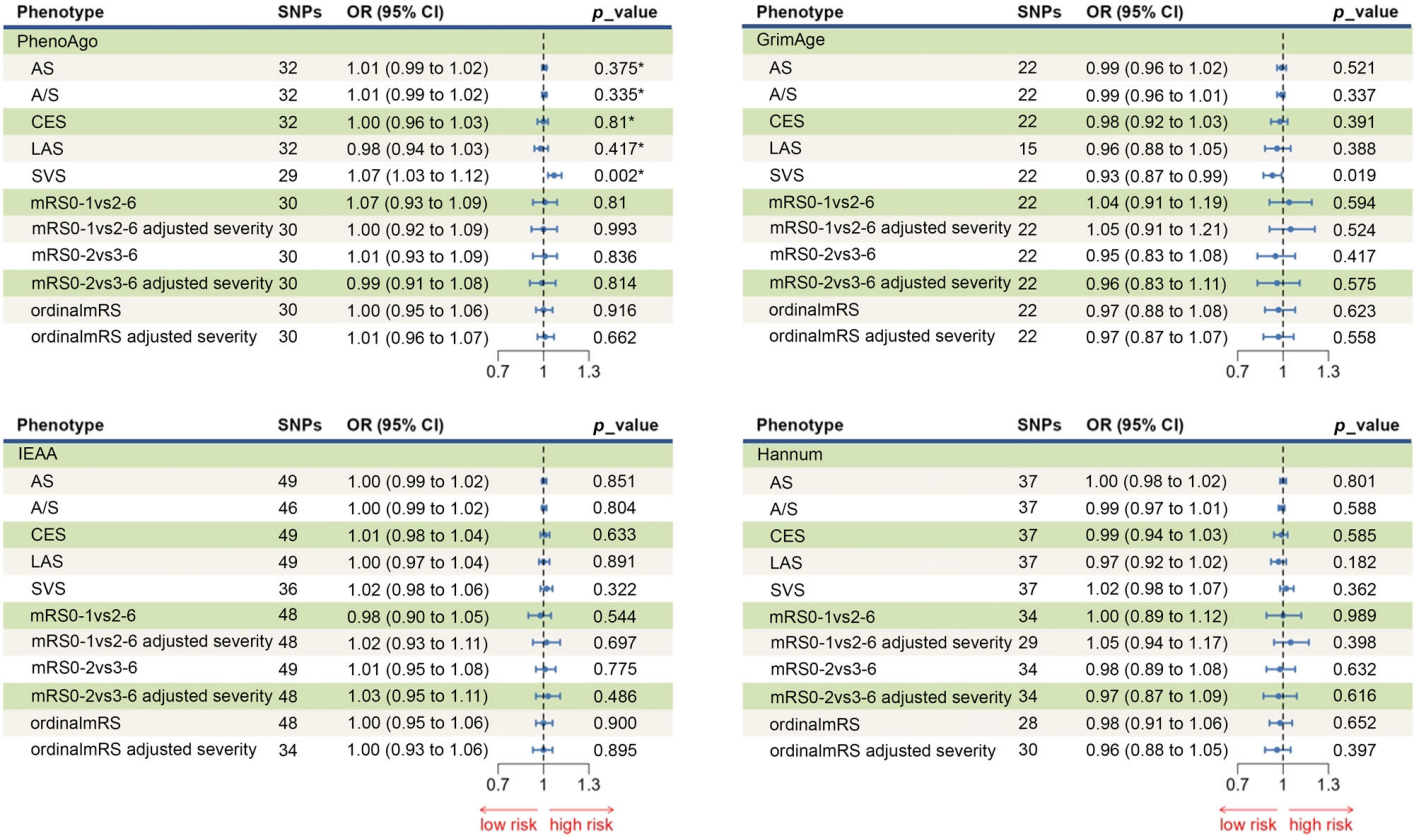
Forest plot for the causal effect of epigenetic age on the risk of stroke and its functional outcome. *It survived after FDR correction.

We found potential associations between two epigenetic age instruments, PhenoAge and GrimAge, and the risk of stroke (Figure 3). PhenoAge was positively correlated with the risk of SVS, whereas GrimAge was negatively correlated with the risk of SVS. The IVW MR analysis revealed that genetically predicted GrimAge reduced the risk of SVS (odds ratio [OR] = 0.93; 95% CI, 0.87–0.99; *p* = 1.86 × 10^−2^). However, genetically determined PhenoAge increased the risk of SVS (OR = 1.07; 95% CI, 1.03–1.12; *p* = 2.01 × 10^−3^). Both the weighted median and MR‐Egger analyses yielded results in the same direction (Table S4). However, the primary IVW result for GrimAge and SVS did not withstand multiple corrections (FDR *p* = 0.19), unlike the result for PhenoAge and SVS (FDR *p* = 0.04). MR‐Egger pleiotropy tests, with *p* values of > 0.05 for the intercept, provided no evidence of pleiotropic bias in our assessment (Table S5). MR‐PRESSO method results were consistent with the causal results, with no identified outliers (Table S5). The leave‐one‐out method confirmed that no single SNP was responsible for the correlations between PhenoAge and GrimAge and SVS (Figures S1 and S2). *F*‐statistics results indicated that our results were not affected by weak instruments (Table S5). There was insufficient evidence to establish a direct link between stroke and other epigenetic age acceleration. Additionally, no substantial evidence suggested causality between epigenetic acceleration and functional outcomes after ischemic stroke (Figure 3). All IVs used in the two‐sample MR analysis are listed in Tables S2 and S3.

### 3.2 Mediation Analysis

The results for lifestyle factors and SVS indicated that only educational attainment and smoking initiation were associated with SVS (Table 1). Genetically predicted educational attainment (OR = 0.70; 95% CI, 0.53–0.92; *p* = 0.01) and smoking initiation (OR = 1.88; 95% CI, 1.24–2.88; *p* = 2.79 × 10^−3^) were linked to SVS. Two‐step MR results are presented in Table 1, wherein the first step showed that increased educational attainment could decelerate PhenoAge (OR = 0.61; 95% CI, 0.40–0.93; *p* = 0.02), and increased smoking initiation could accelerate PhenoAge (OR = 2.36; 95% CI, 1.25–4.44; *p* = 8.08 × 10^−3^). Other sensitivity analyses have confirmed the robustness of our results (Table S5). The results of the MR Steiger tests indicated the correctness of our study's direction (Table 1). Accelerated PhenoAge was associated with an increased risk of SVS (OR = 1.07; 95% CI, 1.03–1.12; *p* = 2.02 × 10^−3^). Finally, we used PhenoAge to assess the indirect impact of smoking initiation and educational attainment on SVS, with mediation proportions calculated using the Delta method (Table 2). The mediation proportions for PhenoAge in educational attainment and smoking initiation were 9.5% (*p* = 4.4 × 10^−2^) and 11.1% (*p* = 5.6 × 10^−2^), respectively (Table 2).

**TABLE 1:** Two‐step MR.

Method	#SNPs	Beta	Low CI	High Cl	*p* value	Steiger test of directionality	
						Direction	Steiger *p* value
Education on SVS							
IVW	274	−0.36	0.53	0.92	0.011	TRUE	0
Weighted median		−0.27	0.51	1.14	0.193		
MR‐Egger		−0.21	0.26	2.53	0.717		
Smoking initiation on SVS							
IVW	139	0.63	1.24	2.84	0.003	TRUE	1.4145E‐199
Weighted median		0.49	0.94	2.85	0.083		
MR‐Egger		1.10	0.35	25.56	0.316		
Lifetime smoking on SVS							
IVW	96	0.54	1.03	2.85	0.037	TRUE	0
Weighted median		0.52	0.79	3.58	0.180		
MR‐Egger		−0.14	0.13	5.96	0.888		
First step							
Education on PhenoAge							
IVW	332	−0.50	0.02	0.40	0.022	TRUE	4.02912E‐05
Weighted median		−0.31	0.33	0.40	0.326		
MR‐Egger		−1.29	0.13	0.05	0.132		
Smoking initiation on PhenoAge							
IVW	132	0.86	1.25	4.44	0.008	TRUE	5.4588E‐228
Weighted median		1.14	1.24	7.93	0.015		
MR‐Egger		1.90	0.37	120.15	0.198		
Second step							
PhenoAge on SVS							
IVW	29	0.07	1.03	1.12	0.002	TRUE	7.8413E‐168
Weighted median		0.08	1.02	1.16	0.009		
MR‐Egger		0.08	0.96	1.22	0.191		

**TABLE 2:** The mediation effect.

Exposure	Mediator	Total effect *β* (95% CI)	Direct effect A *β* (95% CI)	Direct effect B *β* (95% CI)	Mediation effect *β* (95% CI)	Sobel test *p*	Mediated proportion (%)
							(95% CI)
Smoking initiation	PhenoAge	0.63 (1.24–2.88)	0.86 (1.25–4.44)	0.07 (1.03–1.12)	0.06 (0.01–0.13)	0.044	9.5 (1.6–20.6)
Education	PhenoAge	−0.36 (0.53–0.92)	−0.5 (0.40–0.93)	0.07 (1.03–1.12)	0.04 (−0.07 to −0.004)	0.056	11.1 (1.1–19.4)

*Note*: “Total effect” indicates the effect of smoking initiation on SVS and education on SVS, “direct effect A” indicates the effect of smoking initiation and education on PhenoAge, “direct effect B” indicates the effect of PhenoAge on SVS, and “mediation effect” indicates the effect of smoking initiation on SVS and Education on SVS through PhenoAge. Total effect, direct effect A, and direct effect B were derived by IVW, mediation effect was derived by using the delta method. All statistical tests were two‐sided. *p* < 0.05 was considered significant in Sobel test.

## 4 Discussion

To investigate the relationship between epigenetic age and stroke and its functional outcome, we conducted a two‐sample MR analysis. Our findings revealed an association between PhenoAge and GrimAge, two measures of epigenetic age, and SVS. An elevated risk of SVS was linked to an accelerated PhenoAge, whereas GrimAge showed the opposite result. However, the result of GrimAge on SVS was not significant in FDR correction. On the one hand, this result could be attributed to potential bias resulting from the inadequate GWAS sample size, necessitating the need for future GWAS studies with larger sample sizes to investigate the causal correlation. On the other hand, unknown mechanisms may exist, and further research is required to uncover the potential mechanisms between the two. After excluding unreliable results due to multiple testing, we further explored the relationships between PhenoAge and SVS. Unfortunately, this study did not find an association between epigenetic age acceleration and stroke outcomes. Although some studies showed that biological age using DNAm estimates could independently predict ischemic stroke outcomes and that biological age is more advantageous than chronological age in predicting stroke prognosis, it is not supported by our MR analysis based on the latest GWAS data (Soriano‐Tárraga et al. 2017; Wang et al. 2017).

Evaluating previous research, we observed that PhenoAge and SVS showed associations with lifestyle variables such as drinking, smoking, and education (Harshfield et al. 2021; Kong et al. 2023). We hypothesized that lifestyle‐related changes might influence SVS through their effect on PhenoAge. To explore this hypothesis, we examined the causal relationships between lifestyle factors, PhenoAge, and SVS. After a meticulous screening process, we identified initial smoking as the only exposure suitable for the final MR analysis. However, the direction of the beta coefficient for lifetime smoking and SVS was inconsistent, rendering the results unreliable for inclusion in the mediation analysis. In the case of educational attainment, which appeared to protect against SVS by decelerating epigenetic age in the mediation pathway, the Sobel test yielded nonsignificant results, leading us to conclude that it does not affect SVS through epigenetic age and was, therefore, not included in the mediation analysis.

Following rigorous screening and analysis, only smoking initiation emerged as an exposure suitable for the final MR analysis. This study pioneers the exploration of the relationship between epigenetic age and stroke, including its outcomes, while also establishing a link between smoking and SVS mediated through Phenoage age. Previous related research has already established associations between smoking and SVS through epigenetic age. Numerous observational studies and MR investigations have demonstrated that epigenetic age is influenced by various lifestyle factors and, in turn, impacts various health outcomes (Morales et al. 2022; Faul et al. 2023; Kong et al. 2023; Pan et al. 2023; Kawamura et al. 2024).

Understanding whether epigenetic age can mediate the relationship between other exposures and outcomes requires further exploration. Determining whether epigenetic age acts as a moderator in the relationship between other risk factors and illnesses can provide valuable insights for healthcare treatments, disease prognosis, and our understanding of human aging.

Our review of relevant studies (Lin et al. 2023) revealed no significant association between epigenetic age and SVS. This lack of association may be attributed to data quality issues, as the GWAS data used in those studies consisted of intracerebral hemorrhage and SVS. Therefore, our study incorporated the latest epigenetic age data and a large GWAS dataset on SVS, aiming to clarify the comprehensive role of epigenetic age in the pathogenesis of SVS. Furthermore, we investigated the potential mechanisms through which PhenoAge could increase the risk of SVS. Epigenetic age acceleration significantly contributes to explaining the volumetric burden of white matter hyperintensities, which has been found to be associated with brain aging, an increased risk of stroke, and dementia (Raina et al. 2017; Jimenez‐Balado et al. 2022). Several cross‐sectional studies have shown a positive correlation between PhenoAge and C‐reactive protein level, triglyceride levels, and waist‐to‐hip ratio and a negative correlation with high‐density lipoprotein cholesterol level. These factors have strong associations with stroke. PhenoAge has also been observed to activate proinflammatory pathways, such as that of NF‐kappaB, a transcription factor that plays a crucial role in the postischemic activation of the brain, wherein the inflammatory response can exacerbate ischemic injury (Raina et al. 2017). These explanations provide insights into how PhenoAge might increase the risk of SVS. It is important to note that the pathophysiology, prognosis, and clinical features of SVS are distinct from other acute ischemic cerebrovascular diseases, highlighting the need for tailored diagnostic and therapeutic approaches (Rudilosso et al. 2022). For instance, the predominance of cerebral small vessel disease (CSVD) in SVS, characterized by microvascular structural remodeling and chronic inflammation, may amplify the impact of epigenetic age acceleration on disease progression. This distinction underscores the necessity of developing subtype‐specific interventions targeting epigenetic dysregulation and inflammatory pathways in SVS.

In the mediation analysis, we observed that PhenoAge mediates some of the harm caused by smoking with regard to SVS. A previous study has identified a shared mechanistic link between abnormal smoking‐related DNAm and various cancers (Zhou et al. 2023), which may extend to other diseases. We discovered that PhenoAge acceleration increases the risk of several diseases, in addition to an elevated risk of SVS, such as cardiovascular disease, heart disease, diabetes, and Alzheimer's disease (Roberts et al. 2021; Murthy et al. 2023; F. Zhang et al. 2023). Consequently, smoking may lead to SVS by accelerating PhenoAge and could contribute to other diseases via the same mechanism, thereby indirectly increasing the risk of SVS (Chang and Lin 2023).

Our study involves several limitations. First, we just explored the causal association for four epigenetic age measurements with stroke and its outcome; other evaluation indicators in aging could be used to further investigate the causality. Secondly, the genetic data of our study was only collected in European ancestry, which limited consideration for broader racial diversity. Furthermore, we did not extensively explore the protective role of smoking cessation age in SVS incidence and its potential impact on epigenetic age. Finally, we did not conduct subgroup analyses to explore the association between epigenetic age and SVS in different age groups. Previous clinical studies have identified differences in risk factors, stroke subtypes, severity, and outcomes between younger and older individuals with acute ischemic lacunar stroke (Arboix et al. 2015). This represents an important area for further investigation. However, due to the limitations of our dataset, this aspect was not addressed in the current study.

## 5 Conclusion

This study indicates that PhenoAge acceleration is associated with an increased risk of SVS, and smoking initiation further exacerbates this risk by accelerating PhenoAge. Our results support using epigenetic age as a biomarker to predict stroke occurrence. Although our study contributes to the understanding of the relationship between epigenetic age acceleration and SVS, further validation of these associations is needed to explore their clinical significance. Future research should focus on the role of other epigenetic clocks (such as DunedinPACE) in stroke risk and outcomes and delve deeper into the molecular mechanisms linking epigenetic age acceleration to SVS, particularly the roles of inflammation and vascular aging pathways.

## Author Contributions

**Baizhi Qiu**: writing–original draft. **Shuyang Wen**: writing–original draft. **Zifan Li**: writing–original draft. **Yuxin Cai**: methodology. **Qi Zhang**: methodology. **Yuting Zeng**: methodology. **Shuqi Zheng**: methodology. **Zhishan Lin**: methodology. **Yupeng Xiao**: data curation. **Jihua Zou**: writing–review and editing, visualization, formal analysis, software, resources. **Guozhi Huang**: writing–review and editing, funding acquisition, investigation, conceptualization, validation, supervision. **Qing Zeng**: writing–review and editing, formal analysis, data curation, funding acquisition, project administration.

## Ethics Statement

The authors have nothing to report.

## Consent

The authors have nothing to report.

## Conflicts of Interest

The authors declare no conflicts of interest.

### Peer Review

The peer review history for this article is available at https://publons.com/publon/10.1002/brb3.70412

## Supporting information



Supporting Information

Supporting Information

Supporting Information

Supporting Information

Supporting Information

Supporting Information

Supporting Information

Supporting Information

## Data Availability

All summary data for our research are all publicly available (Table S1), and other information such as data sources and sample size can be found in the Supporting Information. The code for our statistical analyses are publicly available on GitHub: https://github.com.
