# Supplementary figures and images for: Causal Associations of Epigenetic Age Acceleration With Stroke and Its Functional Outcome: A Two‐Sample, Two‐Step Mendelian Randomization Study
Source: Brain Behav. 2025 Mar 18;15(3):e70412. doi: 10.1002/brb3.70412 (PMC11919702; doi:10.1002/brb3.70412)

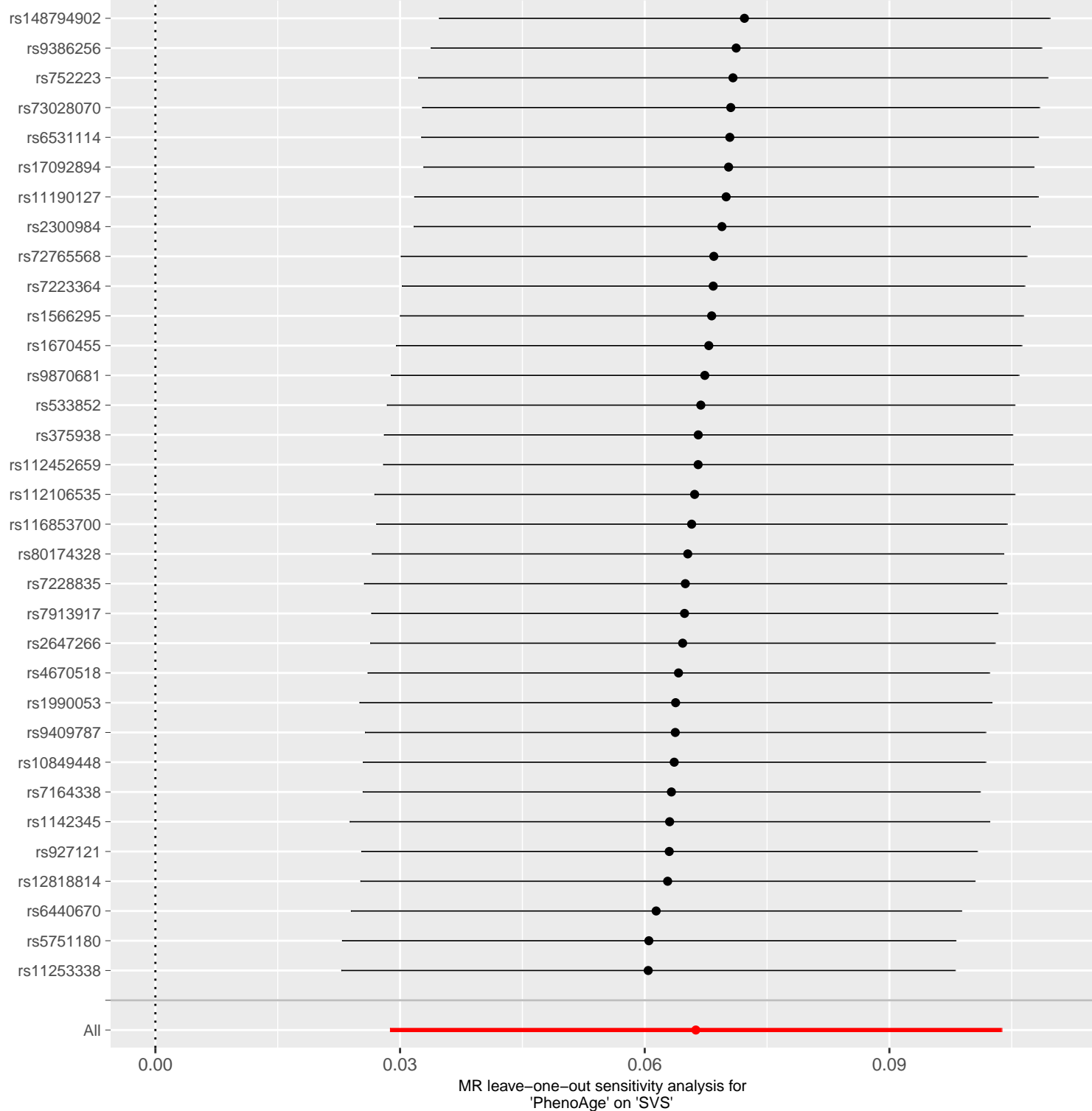

Supplement: Supplementary file 1 — Supporting Information [file BRB3-15-e70412-s001.pdf]

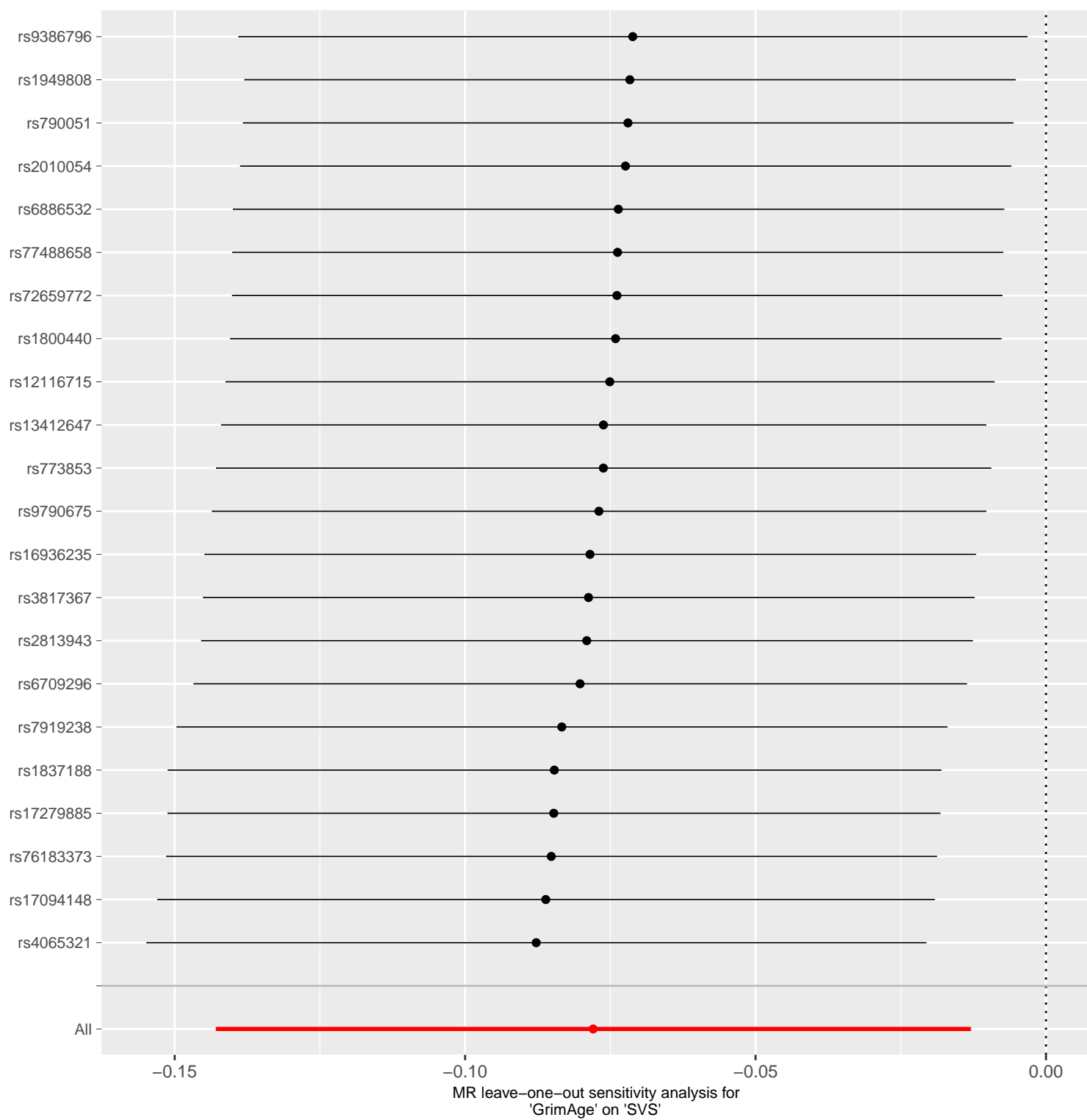

Supplement: Supplementary file 2 — Supporting Information [file BRB3-15-e70412-s002.pdf]
