# Supplementary material for: Causal Associations of Epigenetic Age Acceleration With Stroke and Its Functional Outcome: A Two‐Sample, Two‐Step Mendelian Randomization Study
Source: Brain Behav. 2025 Mar 18;15(3):e70412. doi: 10.1002/brb3.70412 (PMC11919702; doi:10.1002/brb3.70412)
Supplement: Supplementary file 4 — Supporting Information [file BRB3-15-e70412-s005.pdf]

| Detailed data sources | | | | | |
|---|---|---|---|---|---|
| **Phenotypes** | **Trait** | **Consortium** | **Sample Size** | **Population** | **Data sources** |
| Education | Time spent in education measured in years | UKB, 23andMe | 1131881 | European | PMID:30038396 |
| Smoking initiation | included age of initiation of regular smoking and whether an individual had ever smoked regularly | GSCAN | 1232091<br>(557337 cases and 674754controls) | European | PMID:30643251 |
| Lifetime Smoking | a composite of smoking initiation, heaviness, duration and cessation | UKB | 462690 | European | Genome-wide association study of lifetime smoking index in a sample of 462,690 individuals from the UK Biobank |
| Stroke and it's subtypes | included AS, AIS, LAS, CES, SVS | MEGASTROKE | 520000 | European | PMID:29531354 |
| Stroke functional outcome | divided as mRS 0–2 vs 3–6 , mRS 0–1 vs 2–6, and also as the full ordinal scale | GISCOME | 6165 | European | PMID:30796134 |
| epigenetic age | included HannumAge,IEAA,DNAm PhenoAge and DNAm GrimAge | NA | 34710 | European | PMID:8243879 |

any stroke = AS,any ischemic stroke = AIS,large artery stroke = LAS,cardioembolic stroke = CES,small vessel stroke = SVS, intrinsic epigenetic age acceleration (IEAA) is based on the Horvath measure of DNAm age
