# Supplementary material for: Causal Associations of Epigenetic Age Acceleration With Stroke and Its Functional Outcome: A Two‐Sample, Two‐Step Mendelian Randomization Study
Source: Brain Behav. 2025 Mar 18;15(3):e70412. doi: 10.1002/brb3.70412 (PMC11919702; doi:10.1002/brb3.70412)
Supplement: Supplementary file 7 — Supporting Information [file BRB3-15-e70412-s008.pdf]

Main analysis in the univariable mendelian randomization

Exposure	Outcome	#SNPs	MR-Egger			Weighted median			IVW			FDR_P
			P_value	OR	95%CI	P_value	OR	95%CI	P_value	OR	95%CI	
PhenoAge	AS	32	0.512	1.01	0.97 to 1.06	0.491	1.01	0.99 to 1.03	0.375	1.01	0.99 to 1.02	0.758
PhenoAge	AIS	32	0.462	1.02	0.97 to 1.06	0.906	1.00	0.98 to 1.02	0.335	1.01	0.99 to 1.02	0.758
PhenoAge	CES	32	0.663	1.02	0.93 to 1.13	0.805	1.01	0.96 to 1.05	0.810	1.00	0.96 to 1.03	0.891
PhenoAge	LAS	32	0.063	0.89	0.8 to 1	0.295	0.97	0.91 to 1.03	0.417	0.98	0.94 to 1.03	0.758
PhenoAge	SVS	29	0.191	1.08	0.96 to 1.22	0.009	1.09	1.02 to 1.16	0.002	1.07	1.03 to 1.12	0.040
PhenoAge	mRS0-1vs2-6	30	0.516	1.08	0.86 to 1.34	0.485	1.04	0.93 to 1.16	0.810	1.01	0.93 to 1.09	0.993
PhenoAge	mRS0-1vs2-6 adjusted severity	30	0.545	1.08	0.85 to 1.37	0.816	0.99	0.87 to 1.11	0.993	1.00	0.92 to 1.09	0.993
PhenoAge	mRS0-2vs3-6	30	0.780	1.03	0.83 to 1.28	0.760	1.02	0.92 to 1.12	0.836	1.01	0.93 to 1.09	0.993
PhenoAge	mRS0-2vs3-6 adjusted severity	30	0.891	0.98	0.77 to 1.26	0.647	0.97	0.86 to 1.1	0.814	0.99	0.91 to 1.08	0.993
PhenoAge	ordinalmRS	30	0.572	0.96	0.82 to 1.11	0.807	0.99	0.91 to 1.07	0.916	1.00	0.95 to 1.06	0.993
PhenoAge	ordinalmRS adjusted severity	30	0.787	0.98	0.84 to 1.14	0.427	0.97	0.89 to 1.05	0.662	1.01	0.96 to 1.07	0.993
IEAA	AS	49	0.232	1.03	0.98 to 1.07	0.661	1.00	0.98 to 1.03	0.851	1.00	0.99 to 1.02	0.891
IEAA	AIS	46	0.157	1.03	0.99 to 1.08	0.491	1.01	0.99 to 1.03	0.804	1.00	0.99 to 1.02	0.891
IEAA	CES	49	0.776	0.99	0.9 to 1.08	0.977	1.00	0.96 to 1.05	0.633	1.01	0.98 to 1.04	0.844
IEAA	LAS	49	0.908	1.01	0.91 to 1.12	0.676	1.01	0.96 to 1.07	0.891	1.00	0.97 to 1.04	0.891
IEAA	SVS	36	0.031	1.13	1.02 to 1.25	0.495	1.02	0.96 to 1.08	0.322	1.02	0.98 to 1.06	0.758
IEAA	mRS0-1vs2-6	48	0.085	0.83	0.67 to 1.02	0.175	0.93	0.84 to 1.03	0.544	0.98	0.9 to 1.05	0.993
IEAA	mRS0-1vs2-6 adjusted severity	48	0.407	0.90	0.7 to 1.15	0.285	0.94	0.83 to 1.06	0.697	1.02	0.93 to 1.11	0.993
IEAA	mRS0-2vs3-6	49	0.634	0.96	0.8 to 1.15	0.487	0.97	0.88 to 1.06	0.775	1.01	0.95 to 1.08	0.993
IEAA	mRS0-2vs3-6 adjusted severity	48	0.432	0.92	0.74 to 1.14	0.981	1.00	0.9 to 1.12	0.486	1.03	0.95 to 1.11	0.993
IEAA	ordinalmRS	48	0.311	1.08	0.93 to 1.26	0.529	0.98	0.9 to 1.05	0.900	1.00	0.95 to 1.06	0.993
IEAA	ordinalmRS adjusted severity	34	0.329	1.09	0.92 to 1.3	0.274	1.05	0.96 to 1.15	0.895	1.00	0.93 to 1.06	0.993
Hannum	AS	37	0.374	0.97	0.92 to 1.03	0.949	1.00	0.97 to 1.03	0.801	1.00	0.98 to 1.02	0.891
Hannum	AIS	37	0.356	0.97	0.91 to 1.03	0.751	1.00	0.97 to 1.02	0.588	0.99	0.97 to 1.01	0.839
Hannum	CES	37	0.347	0.93	0.81 to 1.08	0.168	0.96	0.9 to 1.02	0.585	0.99	0.94 to 1.03	0.839
Hannum	LAS	37	0.768	0.98	0.83 to 1.15	0.979	1.00	0.93 to 1.08	0.182	0.97	0.92 to 1.02	0.758
Hannum	SVS	37	0.414	1.06	0.92 to 1.22	0.492	1.02	0.96 to 1.09	0.362	1.02	0.98 to 1.07	0.758
Hannum	mRS0-1vs2-6	34	0.618	1.09	0.78 to 1.52	0.327	1.07	0.93 to 1.24	0.989	1.00	0.89 to 1.12	0.993
Hannum	mRS0-1vs2-6 adjusted severity	29	0.117	1.30	0.95 to 1.79	0.267	1.09	0.94 to 1.27	0.398	1.05	0.94 to 1.17	0.993
Hannum	mRS0-2vs3-6	34	0.473	0.90	0.67 to 1.2	0.962	1.00	0.88 to 1.14	0.632	0.98	0.89 to 1.08	0.993
Hannum	mRS0-2vs3-6 adjusted severity	34	0.594	0.91	0.66 to 1.27	0.738	0.98	0.84 to 1.13	0.616	0.97	0.87 to 1.09	0.993
Hannum	ordinalmRS	28	0.652	1.06	0.83 to 1.34	0.649	0.98	0.88 to 1.09	0.652	0.98	0.91 to 1.06	0.993
Hannum	ordinalmRS adjusted severity	30	0.540	0.92	0.7 to 1.2	0.581	0.97	0.86 to 1.09	0.397	0.96	0.88 to 1.05	0.993
GrimAge	AS	22	0.382	0.96	0.87 to 1.06	0.968	1.00	0.96 to 1.04	0.521	0.99	0.96 to 1.02	0.839
GrimAge	AIS	22	0.229	0.92	0.81 to 1.05	0.875	1.00	0.96 to 1.04	0.337	0.99	0.96 to 1.01	0.758
GrimAge	CES	22	0.700	0.95	0.74 to 1.23	0.225	0.95	0.88 to 1.03	0.391	0.98	0.92 to 1.03	0.758
GrimAge	LAS	15	0.255	0.79	0.53 to 1.17	0.685	0.98	0.87 to 1.1	0.388	0.96	0.88 to 1.05	0.758
GrimAge	SVS	22	0.275	0.84	0.62 to 1.14	0.031	0.91	0.83 to 0.99	0.019	0.93	0.87 to 0.99	0.186
GrimAge	mRS0-1vs2-6	22	0.314	1.38	0.75 to 2.53	0.613	0.95	0.78 to 1.15	0.594	1.04	0.91 to 1.19	0.993
GrimAge	mRS0-1vs2-6 adjusted severity	22	0.296	1.42	0.75 to 2.71	0.750	0.97	0.79 to 1.18	0.524	1.05	0.91 to 1.21	0.993
GrimAge	mRS0-2vs3-6	22	0.444	0.79	0.45 to 1.42	0.620	0.96	0.81 to 1.14	0.417	0.95	0.83 to 1.08	0.993
GrimAge	mRS0-2vs3-6 adjusted severity	22	0.139	0.61	0.33 to 1.14	0.375	0.92	0.76 to 1.11	0.575	0.96	0.83 to 1.11	0.993
GrimAge	ordinalmRS	22	0.952	0.99	0.62 to 1.57	0.978	1.00	0.87 to 1.15	0.623	0.97	0.88 to 1.08	0.993
GrimAge	ordinalmRS adjusted severity	22	0.861	1.04	0.65 to 1.66	0.604	0.96	0.84 to 1.11	0.558	0.97	0.87 to 1.07	0.993
Education	PhenoAge	332	0.132	0.27	0.05 to 1.47	0.326	0.74	0.4 to 1.35	0.022	0.61	0.4 to 0.93	
Education	SVS	#274	0.717	0.81	0.26 to 2.53	0.193	0.77	0.51 to 1.14	0.011	0.70	0.53 to 0.92	
Smoking initiation	PhenoAge	132	0.198	6.71	0.37 to 120.15	0.015	3.14	1.24 to 7.93	0.008	2.36	1.25 to 4.44	
Smoking initiation	SVS	139	0.316	3.00	0.35 to 25.56	0.083	1.64	0.94 to 2.85	0.003	1.88	1.24 to 2.84	
Lifetime smoking	SVS	96	0.888	0.87	0.13 to 5.96	0.180	1.68	0.79 to 3.58	0.037	1.71	1.03 to 2.85	
