# Supplementary material for: Causal Associations of Epigenetic Age Acceleration With Stroke and Its Functional Outcome: A Two‐Sample, Two‐Step Mendelian Randomization Study
Source: Brain Behav. 2025 Mar 18;15(3):e70412. doi: 10.1002/brb3.70412 (PMC11919702; doi:10.1002/brb3.70412)
Supplement: Supplementary file 8 — Supporting Information [file BRB3-15-e70412-s003.pdf]

Sensitivity analyses									
Exposure	Outcome	Heterogeneity test				Pleiotropy test			F-statistic
		MREgger_Q	MREgger_P	IVW_Q	IVW_P	Egger_intercept	P_value	MR-PRESSO_P	
PhenoAge	AS	21.893	0.858	22.027	0.882	-0.002	0.717	0.891	20.665
PhenoAge	AIS	22.506	0.835	22.686	0.860	-0.003	0.674	0.857	20.665
PhenoAge	CES	38.405	0.140	38.810	0.158	-0.008	0.578	0.134	20.665
PhenoAge	LAS	32.112	0.362	35.386	0.269	0.027	0.091	0.264	20.665
PhenoAge	SVS	32.234	0.224	32.303	0.262	-0.004	0.812	0.490	20.646
PhenoAge	mRS0-1vs2-6	31.166	0.310	31.585	0.338	-0.018	0.545	0.358	20.890
PhenoAge	mRS0-1vs2-6 adjusted severity	31.127	0.311	31.603	0.338	-0.022	0.518	0.347	20.890
PhenoAge	mRS0-2vs3-6	33.795	0.208	33.855	0.245	-0.006	0.826	0.230	20.890
PhenoAge	mRS0-2vs3-6 adjusted severity	36.342	0.134	36.346	0.164	0.002	0.955	0.151	20.890
PhenoAge	ordinalmRS	27.517	0.490	27.951	0.521	0.014	0.516	0.518	20.890
PhenoAge	ordinalmRS adjusted severity	27.400	0.497	27.620	0.538	0.010	0.643	0.563	20.890
IEAA	AS	61.275	0.079	63.225	0.069	-0.006	0.227	0.051	25.389
IEAA	AIS	35.895	0.803	37.991	0.761	-0.007	0.155	0.737	25.493
IEAA	CES	63.478	0.055	63.795	0.063	0.005	0.630	0.057	25.389
IEAA	LAS	34.134	0.919	34.140	0.934	-0.001	0.942	0.931	25.389
IEAA	SVS	31.153	0.608	35.253	0.456	-0.026	0.051	0.435	24.005
IEAA	mRS0-1vs2-6	56.931	0.152	60.216	0.111	0.042	0.106	0.120	25.602
IEAA	mRS0-1vs2-6 adjusted severity	58.905	0.096	60.292	0.092	0.031	0.303	0.100	25.602
IEAA	mRS0-2vs3-6	47.151	0.466	47.534	0.492	0.013	0.540	0.515	23.518
IEAA	mRS0-2vs3-6 adjusted severity	50.984	0.284	52.368	0.274	0.029	0.270	0.295	25.602
IEAA	ordinalmRS	53.968	0.196	55.257	0.191	-0.019	0.300	0.203	25.602
IEAA	ordinalmRS adjusted severity	38.425	0.201	39.943	0.189	-0.024	0.269	0.202	26.136
Hannum	AS	34.793	0.478	35.543	0.490	0.005	0.393	0.462	24.219
Hannum	AIS	28.665	0.767	29.307	0.777	0.005	0.428	0.771	24.219
Hannum	CES	47.995	0.071	48.920	0.074	0.013	0.417	0.077	24.219
Hannum	LAS	37.840	0.341	37.860	0.384	-0.002	0.894	0.394	24.219
Hannum	SVS	32.920	0.569	33.232	0.601	-0.009	0.580	0.612	24.219
Hannum	mRS0-1vs2-6	45.976	0.052	46.397	0.061	-0.020	0.592	0.066	24.204
Hannum	mRS0-1vs2-6 adjusted severity	17.893	0.907	19.884	0.869	-0.049	0.170	0.872	24.697
Hannum	mRS0-2vs3-6	40.143	0.153	40.595	0.170	0.019	0.553	0.175	24.204
Hannum	mRS0-2vs3-6 adjusted severity	40.948	0.133	41.146	0.156	0.014	0.697	0.159	24.204
Hannum	ordinalmRS	28.854	0.318	29.319	0.346	-0.017	0.523	0.370	24.572
Hannum	ordinalmRS adjusted severity	41.160	0.052	41.342	0.064	0.010	0.728	0.076	23.996
GrimAge	AS	23.286	0.275	23.933	0.296	0.007	0.465	0.312	25.302
GrimAge	AIS	20.162	0.448	21.287	0.442	0.013	0.303	0.415	25.302
GrimAge	CES	18.438	0.559	18.483	0.618	0.005	0.834	0.634	25.302
GrimAge	LAS	8.868	0.783	9.922	0.768	0.040	0.324	0.777	24.177
GrimAge	SVS	9.584	0.975	9.979	0.979	0.018	0.537	0.973	25.302
GrimAge	mRS0-1vs2-6	21.483	0.369	22.428	0.375	-0.056	0.360	0.362	25.302
GrimAge	mRS0-1vs2-6 adjusted severity	18.001	0.587	18.913	0.591	-0.060	0.351	0.586	25.302
GrimAge	mRS0-2vs3-6	24.721	0.212	25.187	0.239	0.035	0.547	0.252	25.302
GrimAge	mRS0-2vs3-6 adjusted severity	21.570	0.364	23.834	0.301	0.088	0.163	0.303	25.302
GrimAge	ordinalmRS	24.375	0.226	24.378	0.275	-0.002	0.961	0.272	25.302
GrimAge	ordinalmRS adjusted severity	22.720	0.303	22.832	0.353	-0.014	0.756	0.385	25.302
Education	PhenoAge	371.640	0.057	372.674	0.057	0.010	0.339	0.066	608.896
Education	SVS	411.259	0.108	411.521	0.113	0.003	0.624	0.802	599.974
Smoking initiation	PhenoAge	108.081	0.920	108.612	0.924	-0.013	0.468	0.930	480.240
Smoking initiation	SVS	161.515	0.075	161.741	0.082	-0.006	0.662	0.075	482.445
Lifetime smoking	SVS	82.159	0.803	82.672	0.813	0.007	0.476	0.808	624.704
