# Supplementary material for: Causal Associations of Epigenetic Age Acceleration With Stroke and Its Functional Outcome: A Two‐Sample, Two‐Step Mendelian Randomization Study
Source: Brain Behav. 2025 Mar 18;15(3):e70412. doi: 10.1002/brb3.70412 (PMC11919702; doi:10.1002/brb3.70412)
Supplement: Supplementary file 6 — Supporting Information [file BRB3-15-e70412-s006.pdf]

Supplemental table 3: Instruments used in the univariable mendelian randomization investigating the effects of epigenetic age on stroke functional outcome

Exposure	Outcome	#SNPs	effect_allele.exposure	other_allele.exposure	beta.exposure	eaf.exposure	se.exposure	pval.exposure
PhenoAge	mRS0-1vs2-6	rs10849448	A	G	0.2346	0.2585	0.048	1.05E-06
		rs11190127	A	C	0.2484	0.3786	0.0397	3.83E-10
		rs11253338	T	C	0.2846	0.184	0.0494	8.49E-09
		rs1142345	T	C	0.8235	0.9454	0.0871	3.35E-21
		rs116853700	A	G	0.5517	0.0425	0.0976	1.59E-08
		rs12818814	T	C	0.2944	0.126	0.0588	5.58E-07
		rs148794902	A	G	-0.2109	0.2656	0.0436	1.34E-06
		rs1566295	T	C	0.179	0.5602	0.0384	3.22E-06
		rs1670455	A	G	-0.2007	0.6935	0.0423	2.11E-06
		rs17092894	T	C	-0.1961	0.29	0.043	4.97E-06
		rs1990053	A	G	0.2573	0.4231	0.0384	2.05E-11
		rs2300984	A	G	-0.1833	0.5757	0.0381	1.54E-06
		rs2647266	A	G	-0.176	0.5922	0.0378	3.28E-06
		rs375938	A	G	-0.1904	0.4179	0.0388	9.22E-07
		rs3829957	T	C	-0.3796	0.1974	0.0482	3.51E-15
		rs4670518	A	C	0.1927	0.2734	0.0422	4.92E-06
		rs533852	T	C	-0.1741	0.4967	0.0375	3.51E-06
		rs5751180	T	C	-0.3915	0.8677	0.0764	3.04E-07
		rs6440670	A	G	-0.2251	0.7966	0.0472	1.90E-06
		rs6531114	T	C	-0.2542	0.2633	0.0424	2.07E-09
		rs678553	T	C	0.3265	0.6892	0.0412	2.27E-15
		rs7164338	T	C	0.2031	0.7274	0.0435	3.07E-06
		rs7223364	T	G	-0.2757	0.122	0.0592	3.21E-06
		rs7228835	C	G	-0.5144	0.1217	0.061	3.30E-17
		rs73028070	A	G	-0.4329	0.0786	0.0768	1.74E-08
		rs752223	A	G	-0.5602	0.0818	0.072	7.20E-15
		rs7913917	T	C	0.1874	0.5625	0.0387	1.29E-06
		rs927121	T	C	0.2149	0.2427	0.0462	3.21E-06
		rs9386256	T	G	-0.372	0.0857	0.0723	2.65E-07
		rs9870681	T	C	0.2221	0.2457	0.0431	2.64E-07
PhenoAge	mRS0-1vs2-6 adjusted severity	rs10849448	A	G	0.2346	0.2585	0.048	1.05E-06
		rs11190127	A	C	0.2484	0.3786	0.0397	3.83E-10
		rs11253338	T	C	0.2846	0.184	0.0494	8.49E-09
		rs1142345	T	C	0.8235	0.9454	0.0871	3.35E-21
		rs116853700	A	G	0.5517	0.0425	0.0976	1.59E-08
		rs12818814	T	C	0.2944	0.126	0.0588	5.58E-07
		rs148794902	A	G	-0.2109	0.2656	0.0436	1.34E-06
		rs1566295	T	C	0.179	0.5602	0.0384	3.22E-06
		rs1670455	A	G	-0.2007	0.6935	0.0423	2.11E-06
		rs17092894	T	C	-0.1961	0.29	0.043	4.97E-06
		rs1990053	A	G	0.2573	0.4231	0.0384	2.05E-11
		rs2300984	A	G	-0.1833	0.5757	0.0381	1.54E-06
		rs2647266	A	G	-0.176	0.5922	0.0378	3.28E-06
		rs375938	A	G	-0.1904	0.4179	0.0388	9.22E-07
		rs3829957	T	C	-0.3796	0.1974	0.0482	3.51E-15
		rs4670518	A	C	0.1927	0.2734	0.0422	4.92E-06
		rs533852	T	C	-0.1741	0.4967	0.0375	3.51E-06
		rs5751180	T	C	-0.3915	0.8677	0.0764	3.04E-07
		rs6440670	A	G	-0.2251	0.7966	0.0472	1.90E-06
		rs6531114	T	C	-0.2542	0.2633	0.0424	2.07E-09
		rs678553	T	C	0.3265	0.6892	0.0412	2.27E-15
		rs7164338	T	C	0.2031	0.7274	0.0435	3.07E-06
		rs7223364	T	G	-0.2757	0.122	0.0592	3.21E-06
		rs7228835	C	G	-0.5144	0.1217	0.061	3.30E-17
		rs73028070	A	G	-0.4329	0.0786	0.0768	1.74E-08
		rs752223	A	G	-0.5602	0.0818	0.072	7.20E-15
		rs7913917	T	C	0.1874	0.5625	0.0387	1.29E-06
		rs927121	T	C	0.2149	0.2427	0.0462	3.21E-06
		rs9386256	T	G	-0.372	0.0857	0.0723	2.65E-07
		rs9870681	T	C	0.2221	0.2457	0.0431	2.64E-07
PhenoAge	mRS0-2vs3-6	rs10849448	A	G	0.2346	0.2585	0.048	1.05E-06
		rs11190127	A	C	0.2484	0.3786	0.0397	3.83E-10
		rs11253338	T	C	0.2846	0.184	0.0494	8.49E-09
		rs1142345	T	C	0.8235	0.9454	0.0871	3.35E-21
		rs116853700	A	G	0.5517	0.0425	0.0976	1.59E-08
		rs12818814	T	C	0.2944	0.126	0.0588	5.58E-07
		rs148794902	A	G	-0.2109	0.2656	0.0436	1.34E-06
		rs1566295	T	C	0.179	0.5602	0.0384	3.22E-06
		rs1670455	A	G	-0.2007	0.6935	0.0423	2.11E-06
		rs17092894	T	C	-0.1961	0.29	0.043	4.97E-06
		rs1990053	A	G	0.2573	0.4231	0.0384	2.05E-11
		rs2300984	A	G	-0.1833	0.5757	0.0381	1.54E-06
		rs2647266	A	G	-0.176	0.5922	0.0378	3.28E-06
		rs375938	A	G	-0.1904	0.4179	0.0388	9.22E-07
		rs3829957	T	C	-0.3796	0.1974	0.0482	3.51E-15
		rs4670518	A	C	0.1927	0.2734	0.0422	4.92E-06
		rs533852	T	C	-0.1741	0.4967	0.0375	3.51E-06
		rs5751180	T	C	-0.3915	0.8677	0.0764	3.04E-07
		rs6440670	A	G	-0.2251	0.7966	0.0472	1.90E-06
		rs6531114	T	C	-0.2542	0.2633	0.0424	2.07E-09
		rs678553	T	C	0.3265	0.6892	0.0412	2.27E-15
		rs7164338	T	C	0.2031	0.7274	0.0435	3.07E-06
		rs7223364	T	G	-0.2757	0.122	0.0592	3.21E-06
		rs7228835	C	G	-0.5144	0.1217	0.061	3.30E-17
		rs73028070	A	G	-0.4329	0.0786	0.0768	1.74E-08
		rs752223	A	G	-0.5602	0.0818	0.072	7.20E-15
		rs7913917	T	C	0.1874	0.5625	0.0387	1.29E-06
		rs927121	T	C	0.2149	0.2427	0.0462	3.21E-06
		rs9386256	T	G	-0.372	0.0857	0.0723	2.65E-07
		rs9870681	T	C	0.2221	0.2457	0.0431	2.64E-07
PhenoAge	mRS0-2vs3-6 adjusted severity	rs10849448	A	G	0.2346	0.2585	0.048	1.05E-06
		rs11190127	A	C	0.2484	0.3786	0.0397	3.83E-10
		rs11253338	T	C	0.2846	0.184	0.0494	8.49E-09
		rs1142345	T	C	0.8235	0.9454	0.0871	3.35E-21
		rs116853700	A	G	0.5517	0.0425	0.0976	1.59E-08
		rs12818814	T	C	0.2944	0.126	0.0588	5.58E-07
		rs148794902	A	G	-0.2109	0.2656	0.0436	1.34E-06
		rs1566295	T	C	0.179	0.5602	0.0384	3.22E-06
		rs1670455	A	G	-0.2007	0.6935	0.0423	2.11E-06
		rs17092894	T	C	-0.1961	0.29	0.043	4.97E-06
		rs1990053	A	G	0.2573	0.4231	0.0384	2.05E-11
		rs2300984	A	G	-0.1833	0.5757	0.0381	1.54E-06
		rs2647266	A	G	-0.176	0.5922	0.0378	3.28E-06
		rs375938	A	G	-0.1904	0.4179	0.0388	9.22E-07
		rs3829957	T	C	-0.3796	0.1974	0.0482	3.51E-15
		rs4670518	A	C	0.1927	0.2734	0.0422	4.92E-06
		rs533852	T	C	-0.1741	0.4967	0.0375	3.51E-06
		rs5751180	T	C	-0.3915	0.8677	0.0764	3.04E-07
		rs6440670	A	G	-0.2251	0.7966	0.0472	1.90E-06
		rs6531114	T	C	-0.2542	0.2633	0.0424	2.07E-09
		rs678553	T	C	0.3265	0.6892	0.0412	2.27E-15
		rs7164338	T	C	0.2031	0.7274	0.0435	3.07E-06
		rs7223364	T	G	-0.2757	0.122	0.0592	3.21E-06
		rs7228835	C	G	-0.5144	0.1217	0.061	3.30E-17
		rs73028070	A	G	-0.4329	0.0786	0.0768	1.74E-08
		rs752223	A	G	-0.5602	0.0818	0.072	7.20E-15
		rs7913917	T	C	0.1874	0.5625	0.0387	1.29E-06
		rs927121	T	C	0.2149	0.2427	0.0462	3.21E-06
		rs9386256	T	G	-0.372	0.0857	0.0723	2.65E-07
		rs9870681	T	C	0.2221	0.2457	0.0431	2.64E-07
PhenoAge	ordinalmRS	rs10849448	A	G	0.2346	0.2585	0.048	1.05E-06
		rs11190127	A	C	0.2484	0.3786	0.0397	3.83E-10
		rs11253338	T	C	0.2846	0.184	0.0494	8.49E-09
		rs1142345	T	C	0.8235	0.9454	0.0871	3.35E-21
		rs116853700	A	G	0.5517	0.0425	0.0976	1.59E-08
		rs12818814	T	C	0.2944	0.126	0.0588	5.58E-07
		rs148794902	A	G	-0.2109	0.2656	0.0436	1.34E-06
		rs1566295	T	C	0.179	0.5602	0.0384	3.22E-06
		rs1670455	A	G	-0.2007	0.6935	0.0423	2.11E-06
		rs17092894	T	C	-0.1961	0.29	0.043	4.97E-06
		rs1990053	A	G	0.2573	0.4231	0.0384	2.05E-11
		rs2300984	A	G	-0.1833	0.5757	0.0381	1.54E-06
		rs2647266	A	G	-0.176	0.5922	0.0378	3.28E-06
		rs375938	A	G	-0.1904	0.4179	0.0388	9.22E-07
		rs3829957	T	C	-0.3796	0.1974	0.0482	3.51E-15
		rs4670518	A	C	0.1927	0.2734	0.0422	4.92E-06
		rs533852	T	C	-0.1741	0.4967	0.0375	3.51E-06
		rs5751180	T	C	-0.3915	0.8677	0.0764	3.04E-07
		rs6440670	A	G	-0.2251	0.7966	0.0472	1.90E-06
		rs6531114	T	C	-0.2542	0.2633	0.0424	2.07E-09
		rs678553	T	C	0.3265	0.6892	0.0412	2.27E-15
		rs7164338	T	C	0.2031	0.7274	0.0435	3.07E-06
		rs7223364	T	G	-0.2757	0.122	0.0592	3.21E-06
		rs7228835	C	G	-0.5144	0.1217	0.061	3.30E-17
		rs73028070	A	G	-0.4329	0.0786	0.0768	1.74E-08
		rs752223	A	G	-0.5602	0.0818	0.072	7.20E-15
		rs7913917	T	C	0.1874	0.5625	0.0387	1.29E-06
		rs927121	T	C	0.2149	0.2427	0.0462	3.21E-06
		rs9386256	T	G	-0.372	0.0857	0.0723	2.65E-07
		rs9870681	T	C	0.2221	0.2457	0.0431	2.64E-07
PhenoAge	ordinalmRS adjusted severity	rs10849448	A	G	0.2346	0.2585	0.048	1.05E-06
		rs11190127	A	C	0.2484	0.3786	0.0397	3.83E-10
		rs11253338	T	C	0.2846	0.184	0.0494	8.49E-09
		rs1142345	T	C	0.8235	0.9454	0.0871	3.35E-21
		rs116853700	A	G	0.5517	0.0425	0.0976	1.59E-08
		rs12818814	T	C	0.2944	0.126	0.0588	5.58E-07
		rs148794902	A	G	-0.2109	0.2656	0.0436	1.34E-06
		rs1566295	T	C	0.179	0.5602	0.0384	3.22E-06
		rs1670455	A	G	-0.2007	0.6935	0.0423	2.11E-06
		rs17092894	T	C	-0.1961	0.29	0.043	4.97E-06
		rs1990053	A	G	0.2573	0.4231	0.0384	2.05E-11
		rs2300984	A	G	-0.1833	0.5757	0.0381	1.54E-06
		rs2647266	A	G	-0.176	0.5922	0.0378	3.28E-06
		rs375938	A	G	-0.1904	0.4179	0.0388	9.22E-07
		rs3829957	T	C	-0.3796	0.1974	0.0482	3.51E-15
		rs4670518	A	C	0.1927	0.2734	0.0422	4.92E-06
		rs533852	T	C	-0.1741	0.4967	0.0375	3.51E-06
		rs5751180	T	C	-0.3915	0.8677	0.0764	3.04E-07
		rs6440670	A	G	-0.2251	0.7966	0.0472	1.90E-06
		rs6531114	T	C	-0.2542	0.2633	0.0424	2.07E-09
		rs678553	T	C	0.3265	0.6892	0.0412	2.27E-15
		rs7164338	T	C	0.2031	0.7274	0.0435	3.07E-06
		rs7223364	T	G	-0.2757	0.122	0.0592	3.21E-06
		rs7228835	C	G	-0.5144	0.1217	0.061	3.30E-17
		rs73028070	A	G	-0.4329	0.0786	0.0768	1.74E-08
		rs752223	A	G	-0.5602	0.0818	0.072	7.20E-15
		rs7913917	T	C	0.1874	0.5625	0.0387	1.29E-06
		rs927121	T	C	0.2149	0.2427	0.0462	3.21E-06
		rs9386256	T	G	-0.372	0.0857	0.0723	2.65E-07
		rs9870681	T	C	0.2221	0.2457	0.0431	2.64E-07
IEAA	mRS0-1vs2-6	rs10192769	T	C	-0.169	0.2272	0.0369	4.63E-06
		rs10447389	A	G	-0.2764	0.2687	0.034	4.42E-16
		rs10732882	T	G	-0.2405	0.4059	0.0318	3.68E-14
		rs10735418	T	C	0.1954	0.6278	0.0321	1.14E-09
		rs10949481	A	T	1.0823	0.9468	0.0699	4.56E-54
		rs12043492	T	C	0.2166	0.4212	0.0313	4.35E-12
		rs12316398	A	T	-0.1727	0.7728	0.0378	4.98E-06
		rs12452981	A	G	0.1821	0.7719	0.0364	5.79E-07
		rs12666349	T	C	0.2548	0.8028	0.0436	5.23E-09
		rs12903325	T	G	-0.2216	0.7596	0.0357	5.48E-10
		rs12940887	T	C	-0.1629	0.3649	0.0316	2.52E-07
		rs13283464	A	C	-0.4703	0.9645	0.0863	5.07E-08
		rs142394165	A	G	0.641	0.0235	0.1395	4.33E-06
		rs144317085	A	T	0.5136	0.9612	0.0831	6.42E-10
		rs1488106	T	C	0.1827	0.3721	0.0311	4.25E-09
		rs1511762	T	C	0.2642	0.2186	0.0374	1.53E-12
		rs1569419	T	C	-0.2175	0.2368	0.0425	3.06E-07
		rs1607476	A	C	-0.1662	0.4384	0.0312	1.02E-07
		rs1726672	T	C	-0.2038	0.3036	0.0329	6.05E-10
		rs17667771	A	G	0.3897	0.0721	0.0848	4.34E-06
		rs2070457	A	C	-0.162	0.2931	0.0342	2.16E-06
		rs2275558	A	G	-0.2335	0.2212	0.0402	6.44E-09
		rs2492286	T	G	0.2808	0.1519	0.0428	5.29E-11
		rs2736099	A	G	0.2326	0.3538	0.0334	3.47E-12
		rs2823020	T	G	-0.2766	0.9047	0.0536	2.41E-07
		rs34003787	T	C	0.3239	0.0862	0.0581	2.54E-08
		rs361071	A	G	0.1595	0.4587	0.0299	9.70E-08
		rs3917672	A	G	-0.2622	0.4858	0.0304	5.95E-18
		rs4240228	T	G	0.2547	0.7168	0.0334	2.47E-14
		rs57941717	T	G	0.2908	0.2528	0.0357	3.71E-16
		rs62081212	A	G	-0.3376	0.0631	0.0672	5.01E-07
		rs62130084	T	C	0.4329	0.0438	0.0922	2.68E-06
		rs62405449	T	C	-0.2274	0.8453	0.0481	2.22E-06
		rs6414374	A	G	0.3214	0.1625	0.0418	1.42E-14
		rs6577536	A	G	0.1968	0.4843	0.0302	6.73E-11
		rs6685614	A	G	-0.1638	0.7393	0.0357	4.37E-06
		rs6818931	C	G	0.176	0.254	0.0377	2.97E-06
		rs7202333	T	C	-0.1507	0.6037	0.031	1.17E-06
		rs72928038	A	G	0.201	0.1806	0.0426	2.34E-06
		rs74827125	A	C	-0.2932	0.0812	0.0571	2.77E-07
		rs75243280	T	C	-0.2323	0.6686	0.0339	7.37E-12
		rs75308704	T	C	0.3027	0.9265	0.0627	1.39E-06
		rs7550821	T	C	-0.2551	0.2441	0.035	3.27E-13
		rs7627756	A	G	0.2162	0.5615	0.0301	6.28E-13
		rs7705463	T	C	0.2155	0.1592	0.0426	4.24E-07
		rs7909074	A	G	-0.1542	0.5787	0.0306	4.76E-07
		rs7946876	A	G	-0.2474	0.1014	0.0518	1.76E-06
		rs9301758	T	C	0.1493	0.5228	0.0317	2.57E-06
		rs9636077	A	C	-0.1546	0.5241	0.0306	4.23E-07
IEAA	mRS0-1vs2-6 adjusted severity	rs10192769	T	C	-0.169	0.2272	0.0369	4.63E-06
		rs10447389	A	G	-0.2764	0.2687	0.034	4.42E-16
		rs10732882	T	G	-0.2405	0.4059	0.0318	3.68E-14
		rs10735418	T	C	0.1954	0.6278	0.0321	1.14E-09
		rs10949481	A	T	1.0823	0.9468	0.0699	4.56E-54
		rs12043492	T	C	0.2166	0.4212	0.0313	4.35E-12
		rs12316398	A	T	-0.1727	0.7728	0.0378	4.98E-06
		rs12452981	A	G	0.1821	0.7719	0.0364	5.79E-07
		rs12666349	T	C	0.2548	0.8028	0.0436	5.23E-09
		rs12903325	T	G	-0.2216	0.7596	0.0357	5.48E-10
		rs12940887	T	C	-0.1629	0.3649	0.0316	2.52E-07
		rs13283464	A	C	-0.4703	0.9645	0.0863	5.07E-08
		rs144317085	A	T	0.5136	0.9612	0.0831	6.42E-10
		rs1488106	T	C	0.1827	0.3721	0.0311	4.25E-09
		rs1511762	T	C	0.2642	0.2186	0.0374	1.53E-12
		rs1569419	T	C	-0.2175	0.2368	0.0425	3.06E-07
		rs1607476	A	C	-0.1662	0.4384	0.0312	1.02E-07
		rs1726672	T	C	-0.2038	0.3036	0.0329	6.05E-10
		rs17667771	A	G	0.3897	0.0721	0.0848	4.34E-06
		rs2070457	A	C	-0.162	0.2931	0.0342	2.16E-06
		rs2275558	A	G	-0.2335	0.2212	0.0402	6.44E-09
		rs2492286	T	G	0.2808	0.1519	0.0428	5.29E-11
		rs2736099	A	G	0.2326	0.3538	0.0334	3.47E-12
		rs2823020	T	G	-0.2766	0.9047	0.0536	2.41E-07
		rs34003787	T	C	0.3239	0.0862	0.0581	2.54E-08
		rs361071	A	G	0.1595	0.4587	0.0299	9.70E-08
		rs3917672	A	G	-0.2622	0.4858	0.0304	5.95E-18
		rs4240228	T	G	0.2547	0.7168	0.0334	2.47E-14
		rs57941717	T	G	0.2908	0.2528	0.0357	3.71E-16
		rs62081212	A	G	-0.3376	0.0631	0.0672	5.01E-07
		rs62130084	T	C	0.4329	0.0438	0.0922	2.68E-06
		rs62405449	T	C	-0.2274	0.8453	0.0481	2.22E-06
		rs6414374	A	G	0.3214	0.1625	0.0418	1.42E-14
		rs6577536	A	G	0.1968	0.4843	0.0302	6.73E-11
		rs6685614	A	G	-0.1638	0.7393	0.0357	4.37E-06
		rs6818931	C	G	0.176	0.254	0.0377	2.97E-06
		rs7202333	T	C	-0.1507	0.6037	0.031	1.17E-06
		rs72928038	A	G	0.201	0.1806	0.0426	2.34E-06
		rs74827125	A	C	-0.2932	0.0812	0.0571	2.77E-07
		rs75243280	T	C	-0.2323	0.6686	0.0339	7.37E-12
		rs75308704	T	C	0.3027	0.9265	0.0627	1.39E-06
		rs7550821	T	C	-0.2551	0.2441	0.035	3.27E-13
		rs7627756	A	G	0.2162	0.5615	0.0301	6.28E-13
		rs7705463	T	C	0.2155	0.1592	0.0426	4.24E-07
		rs7909074	A	G	-0.1542	0.5787	0.0306	4.76E-07
		rs7946876	A	G	-0.2474	0.1014	0.0518	1.76E-06
		rs9301758	T	C	0.1493	0.5228	0.0317	2.57E-06
		rs9636077	A	C	-0.1546	0.5241	0.0306	4.23E-07
IEAA	mRS0-2vs3-6	rs10192769	T	C	-0.169	0.2272	0.0369	4.63E-06
		rs10447389	A	G	-0.2764	0.2687	0.034	4.42E-16
		rs10732882	T	G	-0.2405	0.4059	0.0318	3.68E-14
		rs10735418	T	C	0.1954	0.6278	0.0321	1.14E-09
		rs10949481	A	T	1.0823	0.9468	0.0699	4.56E-54
		rs11762078	T	G	0.4634	0.0355	0.1009	4.39E-06
		rs12043492	T	C	0.2166	0.4212	0.0313	4.35E-12
		rs12316398	A	T	-0.1727	0.7728	0.0378	4.98E-06
		rs12452981	A	G	0.1821	0.7719	0.0364	5.79E-07
		rs12666349	T	C	0.2548	0.8028	0.0436	5.23E-09
		rs12903325	T	G	-0.2216	0.7596	0.0357	5.48E-10
		rs12940887	T	C	-0.1629	0.3649	0.0316	2.52E-07
		rs13283464	A	C	-0.4703	0.9645	0.0863	5.07E-08
		rs144317085	A	T	0.5136	0.9612	0.0831	6.42E-10
		rs1488106	T	C	0.1827	0.3721	0.0311	4.25E-09
		rs1511762	T	C	0.2642	0.2186	0.0374	1.53E-12
		rs1569419	T	C	-0.2175	0.2368	0.0425	3.06E-07
		rs1607476	A	C	-0.1662	0.4384	0.0312	1.02E-07
		rs1726672	T	C	-0.2038	0.3036	0.0329	6.05E-10
		rs17667771	A	G	0.3897	0.0721	0.0848	4.34E-06
		rs2070457	A	C	-0.162	0.2931	0.0342	2.16E-06
		rs2275558	A	G	-0.2335	0.2212	0.0402	6.44E-09
		rs2492286	T	G	0.2808	0.1519	0.0428	5.29E-11
		rs2736099	A	G	0.2326	0.3538	0.0334	3.47E-12
		rs2823020	T	G	-0.2766	0.9047	0.0536	2.41E-07
		rs34003787	T	C	0.3239	0.0862	0.0581	2.54E-08
		rs361071	A	G	0.1595	0.4587	0.0299	9.70E-08
		rs3917672	A	G	-0.2622	0.4858	0.0304	5.95E-18
		rs4240228	T	G	0.2547	0.7168	0.0334	2.47E-14
		rs57941717	T	G	0.2908	0.2528	0.0357	3.71E-16
		rs62081212	A	G	-0.3376	0.0631	0.0672	5.01E-07
		rs62130084	T	C	0.4329	0.0438	0.0922	2.68E-06
		rs62405449	T	C	-0.2274	0.8453	0.0481	2.22E-06
		rs6414374	A	G	0.3214	0.1625	0.0418	1.42E-14
		rs6577536	A	G	0.1968	0.4843	0.0302	6.73E-11
		rs6685614	A	G	-0.1638	0.7393	0.0357	4.37E-06
		rs6818931	C	G	0.176	0.254	0.0377	2.97E-06
		rs7202333	T	C	-0.1507	0.6037	0.031	1.17E-06
		rs72928038	A	G	0.201	0.1806	0.0426	2.34E-06
		rs74827125	A	C	-0.2932	0.0812	0.0571	2.77E-07
		rs75243280	T	C	-0.2323	0.6686	0.0339	7.37E-12
		rs75308704	T	C	0.3027	0.9265	0.0627	1.39E-06
		rs7550821	T	C	-0.2551	0.2441	0.035	3.27E-13
		rs7627756	A	G	0.2162	0.5615	0.0301	6.28E-13
		rs7705463	T	C	0.2155	0.1592	0.0426	4.24E-07
		rs7909074	A	G	-0.1542	0.5787	0.0306	4.76E-07
		rs7946876	A	G	-0.2474	0.1014	0.0518	1.76E-06
		rs9301758	T	C	0.1493	0.5228	0.0317	2.57E-06
		rs9636077	A	C	-0.1546	0.5241	0.0306	4.23E-07
IEAA	mRS0-2vs3-6 adjusted severity	rs10192769	T	C	-0.169	0.2272	0.0369	4.63E-06
		rs10447389	A	G	-0.2764	0.2687	0.034	4.42E-16
		rs10732882	T	G	-0.2405	0.4059	0.0318	3.68E-14
		rs10735418	T	C	0.1954	0.6278	0.0321	1.14E-09
		rs10949481	A	T	1.0823	0.9468	0.0699	4.56E-54
		rs12043492	T	C	0.2166	0.4212	0.0313	4.35E-12
		rs12316398	A	T	-0.1727	0.7728	0.0378	4.98E-06
		rs12452981	A	G	0.1821	0.7719	0.0364	5.79E-07
		rs12666349	T	C	0.2548	0.8028	0.0436	5.23E-09
		rs12903325	T	G	-0.2216	0.7596	0.0357	5.48E-10
		rs12940887	T	C	-0.1629	0.3649	0.0316	2.52E-07
		rs13283464	A	C	-0.4703	0.9645	0.0863	5.07E-08
		rs144317085	A	T	0.5136	0.9612	0.0831	6.42E-10
		rs1488106	T	C	0.1827	0.3721	0.0311	4.25E-09
		rs1511762	T	C	0.2642	0.2186	0.0374	1.53E-12
		rs1569419	T	C	-0.2175	0.2368	0.0425	3.06E-07
		rs1607476	A	C	-0.1662	0.4384	0.0312	1.02E-07
		rs1726672	T	C	-0.2038	0.3036	0.0329	6.05E-10
		rs17667771	A	G	0.3897	0.0721	0.0848	4.34E-06
		rs2070457	A	C	-0.162	0.2931	0.0342	2.16E-06
		rs2275558	A	G	-0.2335	0.2212	0.0402	6.44E-09
		rs2492286	T	G	0.2808	0.1519	0.0428	5.29E-11
		rs2736099	A	G	0.2326	0.3538	0.0334	3.47E-12
		rs2823020	T	G	-0.2766	0.9047	0.0536	2.41E-07
		rs34003787	T	C	0.3239	0.0862	0.0581	2.54E-08
		rs361071	A	G	0.1595	0.4587	0.0299	9.70E-08
		rs3917672	A	G	-0.2622	0.4858	0.0304	5.95E-18
		rs4240228	T	G	0.2547	0.7168	0.0334	2.47E-14
		rs57941717	T	G	0.2908	0.2528	0.0357	3.71E-16
		rs62081212	A	G	-0.3376	0.0631	0.0672	5.01E-07
		rs62130084	T	C	0.4329	0.0438	0.0922	2.68E-06
		rs62405449	T	C	-0.2274	0.8453	0.0481	2.22E-06
		rs6414374	A	G	0.3214	0.1625	0.0418	1.42E-14
		rs6577536	A	G	0.1968	0.4843	0.0302	6.73E-11
		rs6685614	A	G	-0.1638	0.7393	0.0357	4.37E-06
		rs6818931	C	G	0.176	0.254	0.0377	2.97E-06
		rs7202333	T	C	-0.1507	0.6037	0.031	1.17E-06
		rs72928038	A	G	0.201	0.1806	0.0426	2.34E-06
		rs74827125	A	C	-0.2932	0.0812	0.0571	2.77E-07
		rs75243280	T	C	-0.2323	0.6686	0.0339	7.37E-12
		rs75308704	T	C	0.3027	0.9265	0.0627	1.39E-06
		rs7550821	T	C	-0.2551	0.2441	0.035	3.27E-13
		rs7627756	A	G	0.2162	0.5615	0.0301	6.28E-13
		rs7705463	T	C	0.2155	0.1592	0.0426	4.24E-07
		rs7909074	A	G	-0.1542	0.5787	0.0306	4.76E-07
		rs7946876	A	G	-0.2474	0.1014	0.0518	1.76E-06
		rs9301758	T	C	0.1493	0.5228	0.0317	2.57E-06
		rs9636077	A	C	-0.1546	0.5241	0.0306	4.23E-07
IEAA	ordinalmRS	rs10192769	T	C	-0.169	0.2272	0.0369	4.63E-06
		rs10447389	A	G	-0.2764	0.2687	0.034	4.42E-16
		rs10732882	T	G	-0.2405	0.4059	0.0318	3.68E-14
		rs10735418	T	C	0.1954	0.6278	0.0321	1.14E-09
		rs10949481	A	T	1.0823	0.9468	0.0699	4.56E-54
		rs12043492	T	C	0.2166	0.4212	0.0313	4.35E-12
		rs12316398	A	T	-0.1727	0.7728	0.0378	4.98E-06
		rs12452981	A	G	0.1821	0.7719	0.0364	5.79E-07
		rs12666349	T	C	0.2548	0.8028	0.0436	5.23E-09
		rs12903325	T	G	-0.2216	0.7596	0.0357	5.48E-10
		rs12940887	T	C	-0.1629	0.3649	0.0316	2.52E-07
		rs13283464	A	C	-0.4703	0.9645	0.0863	5.07E-08
		rs144317085	A	T	0.5136	0.9612	0.0831	6.42E-10
		rs1488106	T	C	0.1827	0.3721	0.0311	4.25E-09
		rs1511762	T	C	0.2642	0.2186	0.0374	1.53E-12
		rs1569419	T	C	-0.2175	0.2368	0.0425	3.06E-07
		rs1607476	A	C	-0.1662	0.4384	0.0312	1.02E-07
		rs1726672	T	C	-0.2038	0.3036	0.0329	6.05E-10
		rs17667771	A	G	0.3897	0.0721	0.0848	4.34E-06
		rs2070457	A	C	-0.162	0.2931	0.0342	2.16E-06
		rs2275558	A	G	-0.2335	0.2212	0.0402	6.44E-09
		rs2492286	T	G	0.2808	0.1519	0.0428	5.29E-11
		rs2736099	A	G	0.2326	0.3538	0.0334	3.47E-12
		rs2823020	T	G	-0.2766	0.9047	0.0536	2.41E-07
		rs34003787	T	C	0.3239	0.0862	0.0581	2.54E-08
		rs361071	A	G	0.1595	0.4587	0.0299	9.70E-08
		rs3917672	A	G	-0.2622	0.4858	0.0304	5.95E-18
		rs4240228	T	G	0.2547	0.7168	0.0334	2.47E-14
		rs57941717	T	G	0.2908	0.2528	0.0357	3.71E-16
		rs62081212	A	G	-0.3376	0.0631	0.0672	5.01E-07
		rs62130084	T	C	0.4329	0.0438	0.0922	2.68E-06
		rs62405449	T	C	-0.2274	0.8453	0.0481	2.22E-06
		rs6414374	A	G	0.3214	0.1625	0.0418	1.42E-14
		rs6577536	A	G	0.1968	0.4843	0.0302	6.73E-11
		rs6685614	A	G	-0.1638	0.7393	0.0357	4.37E-06
		rs6818931	C	G	0.176	0.254	0.0377	2.97E-06
		rs7202333	T	C	-0.1507	0.6037	0.031	1.17E-06
		rs72928038	A	G	0.201	0.1806	0.0426	2.34E-06
		rs74827125	A	C	-0.2932	0.0812	0.0571	2.77E-07
		rs75243280	T	C	-0.2323	0.6686	0.0339	7.37E-12
		rs75308704	T	C	0.3027	0.9265	0.0627	1.39E-06
		rs7550821	T	C	-0.2551	0.2441	0.035	3.27E-13
		rs7627756	A	G	0.2162	0.5615	0.0301	6.28E-13
		rs7705463	T	C	0.2155	0.1592	0.0426	4.24E-07
		rs7909074	A	G	-0.1542	0.5787	0.0306	4.76E-07
		rs7946876	A	G	-0.2474	0.1014	0.0518	1.76E-06
		rs9301758	T	C	0.1493	0.5228	0.0317	2.57E-06
		rs9636077	A	C	-0.1546	0.5241	0.0306	4.23E-07
IEAA	ordinalmRS adjusted severity	rs10192769	T	C	-0.169	0.2272	0.0369	4.63E-06
		rs10447389	A	G	-0.2764	0.2687	0.034	4.42E-16
		rs10732882	T	G	-0.2405	0.4059	0.0318	3.68E-14
		rs10949481	A	T	1.0823	0.9468	0.0699	4.56E-54
		rs12043492	T	C	0.2166	0.4212	0.0313	4.35E-12
		rs12316398	A	T	-0.1727	0.7728	0.0378	4.98E-06
		rs12452981	A	G	0.1821	0.7719	0.0364	5.79E-07
		rs12666349	T	C	0.2548	0.8028	0.0436	5.23E-09
		rs12903325	T	G	-0.2216	0.7596	0.0357	5.48E-10
		rs13283464	A	C	-0.4703	0.9645	0.0863	5.07E-08
		rs144317085	A	T	0.5136	0.9612	0.0831	6.42E-10
		rs1488106	T	C	0.1827	0.3721	0.0311	4.25E-09
		rs1569419	T	C	-0.2175	0.2368	0.0425	3.06E-07
		rs1726672	T	C	-0.2038	0.3036	0.0329	6.05E-10
		rs2070457	A	C	-0.162	0.2931	0.0342	2.16E-06
		rs2492286	T	G	0.2808	0.1519	0.0428	5.29E-11
		rs2736099	A	G	0.2326	0.3538	0.0334	3.47E-12
		rs2823020	T	G	-0.2766	0.9047	0.0536	2.41E-07
		rs3917672	A	G	-0.2622	0.4858	0.0304	5.95E-18
		rs57941717	T	G	0.2908	0.2528	0.0357	3.71E-16
		rs62405449	T	C	-0.2274	0.8453	0.0481	2.22E-06
		rs6414374	A	G	0.3214	0.1625	0.0418	1.42E-14
		rs6577536	A	G	0.1968	0.4843	0.0302	6.73E-11
		rs6685614	A	G	-0.1638	0.7393	0.0357	4.37E-06
		rs6818931	C	G	0.176	0.254	0.0377	2.97E-06
		rs7202333	T	C	-0.1507	0.6037	0.031	1.17E-06
		rs74827125	A	C	-0.2932	0.0812	0.0571	2.77E-07
		rs75243280	T	C	-0.2323	0.6686	0.0339	7.37E-12
		rs75308704	T	C	0.3027	0.9265	0.0627	1.39E-06
		rs7550821	T	C	-0.2551	0.2441	0.035	3.27E-13
		rs7627756	A	G	0.2162	0.5615	0.0301	6.28E-13
		rs7705463	T	C	0.2155	0.1592	0.0426	4.24E-07
		rs7909074	A	G	-0.1542	0.5787	0.0306	4.76E-07
		rs9301758	T	C	0.1493	0.5228	0.0317	2.57E-06
Hannum	mRS0-1vs2-6	rs1005277	A	C	0.3006	0.2982	0.033	8.92E-20
		rs10786282	A	G	-0.36	0.2124	0.0362	2.61E-23
		rs11190130	T	C	0.147	0.3869	0.0305	1.44E-06
		rs114606172	T	C	0.3554	0.0538	0.0677	1.54E-07
		rs1200332	T	C	0.179	0.2061	0.0371	1.44E-06
		rs12196813	T	C	0.4614	0.0385	0.0963	1.64E-06
		rs12271802	A	G	0.364	0.0552	0.0765	1.95E-06
		rs12417758	T	C	-0.2093	0.5461	0.0304	6.22E-12
		rs139107270	A	T	0.4628	0.0376	0.0959	1.39E-06
		rs148436209	T	C	0.49	0.0334	0.0952	2.64E-07
		rs1598856	A	G	0.1858	0.4492	0.0296	3.63E-10
		rs16958455	T	C	-0.1533	0.6322	0.0314	1.09E-06
		rs1824965	T	C	-0.1456	0.4524	0.0308	2.24E-06
		rs1849209	T	G	-0.1647	0.7591	0.0347	2.07E-06
		rs1984968	A	G	0.2047	0.8451	0.0414	7.44E-07
		rs2014202	A	G	0.1696	0.3246	0.0315	7.23E-08
		rs2195114	T	C	-0.1614	0.3345	0.031	1.94E-07
		rs2229742	C	G	0.241	0.1323	0.0501	1.49E-06
		rs2647254	A	G	-0.1436	0.3504	0.0312	4.03E-06
		rs34970912	C	G	-0.5213	0.9648	0.0939	2.88E-08
		rs4383328	A	T	-0.1889	0.2848	0.0329	9.57E-09
		rs4679900	T	C	0.1538	0.5494	0.0304	4.31E-07
		rs4770473	T	C	0.159	0.4888	0.0306	2.00E-07
		rs4838595	T	C	-0.2576	0.1217	0.0453	1.29E-08
		rs6054829	A	G	-0.276	0.9221	0.0601	4.43E-06
		rs62385557	T	C	0.279	0.0718	0.0597	2.94E-06
		rs6440670	A	G	-0.1915	0.7966	0.0367	1.79E-07
		rs7017774	T	C	0.146	0.506	0.0303	1.44E-06
		rs7705526	A	C	0.1811	0.3428	0.0338	8.52E-08
		rs7965945	T	C	-0.2095	0.8457	0.0424	7.92E-07
		rs8005306	A	C	0.322	0.0539	0.0684	2.51E-06
		rs80195828	T	C	0.2695	0.9189	0.0562	1.59E-06
		rs878175	T	C	-0.1903	0.8105	0.0385	7.49E-07
		rs9813840	T	C	0.4373	0.96	0.0859	3.58E-07
Hannum	mRS0-1vs2-6 adjusted severity	rs1005277	A	C	0.3006	0.2982	0.033	8.92E-20
		rs10786282	A	G	-0.36	0.2124	0.0362	2.61E-23
		rs11190130	T	C	0.147	0.3869	0.0305	1.44E-06
		rs114606172	T	C	0.3554	0.0538	0.0677	1.54E-07
		rs1200332	T	C	0.179	0.2061	0.0371	1.44E-06
		rs12196813	T	C	0.4614	0.0385	0.0963	1.64E-06
		rs12271802	A	G	0.364	0.0552	0.0765	1.95E-06
		rs12417758	T	C	-0.2093	0.5461	0.0304	6.22E-12
		rs139107270	A	T	0.4628	0.0376	0.0959	1.39E-06
		rs148436209	T	C	0.49	0.0334	0.0952	2.64E-07
		rs1598856	A	G	0.1858	0.4492	0.0296	3.63E-10
		rs16958455	T	C	-0.1533	0.6322	0.0314	1.09E-06
		rs1824965	T	C	-0.1456	0.4524	0.0308	2.24E-06
		rs1849209	T	G	-0.1647	0.7591	0.0347	2.07E-06
		rs1984968	A	G	0.2047	0.8451	0.0414	7.44E-07
		rs2014202	A	G	0.1696	0.3246	0.0315	7.23E-08
		rs2195114	T	C	-0.1614	0.3345	0.031	1.94E-07
		rs2229742	C	G	0.241	0.1323	0.0501	1.49E-06
		rs2647254	A	G	-0.1436	0.3504	0.0312	4.03E-06
		rs34970912	C	G	-0.5213	0.9648	0.0939	2.88E-08
		rs4383328	A	T	-0.1889	0.2848	0.0329	9.57E-09
		rs4679900	T	C	0.1538	0.5494	0.0304	4.31E-07
		rs4770473	T	C	0.159	0.4888	0.0306	2.00E-07
		rs4838595	T	C	-0.2576	0.1217	0.0453	1.29E-08
		rs6054829	A	G	-0.276	0.9221	0.0601	4.43E-06
		rs62385557	T	C	0.279	0.0718	0.0597	2.94E-06
		rs6440670	A	G	-0.1915	0.7966	0.0367	1.79E-07
		rs7017774	T	C	0.146	0.506	0.0303	1.44E-06
		rs7705526	A	C	0.1811	0.3428	0.0338	8.52E-08
		rs7965945	T	C	-0.2095	0.8457	0.0424	7.92E-07
		rs8005306	A	C	0.322	0.0539	0.0684	2.51E-06
		rs80195828	T	C	0.2695	0.9189	0.0562	1.59E-06
		rs878175	T	C	-0.1903	0.8105	0.0385	7.49E-07
		rs9813840	T	C	0.4373	0.96	0.0859	3.58E-07
Hannum	mRS0-2vs3-6	rs1005277	A	C	0.3006	0.2982	0.033	8.92E-20
		rs10786282	A	G	-0.36	0.2124	0.0362	2.61E-23
		rs11190130	T	C	0.147	0.3869	0.0305	1.44E-06
		rs114606172	T	C	0.3554	0.0538	0.0677	1.54E-07
		rs1200332	T	C	0.179	0.2061	0.0371	1.44E-06
		rs12196813	T	C	0.4614	0.0385	0.0963	1.64E-06
		rs12271802	A	G	0.364	0.0552	0.0765	1.95E-06
		rs12417758	T	C	-0.2093	0.5461	0.0304	6.22E-12
		rs139107270	A	T	0.4628	0.0376	0.0959	1.39E-06
		rs148436209	T	C	0.49	0.0334	0.0952	2.64E-07
		rs1598856	A	G	0.1858	0.4492	0.0296	3.63E-10
		rs16958455	T	C	-0.1533	0.6322	0.0314	1.09E-06
		rs1824965	T	C	-0.1456	0.4524	0.0308	2.24E-06
		rs1849209	T	G	-0.1647	0.7591	0.0347	2.07E-06
		rs1984968	A	G	0.2047	0.8451	0.0414	7.44E-07
		rs2014202	A	G	0.1696	0.3246	0.0315	7.23E-08
		rs2195114	T	C	-0.1614	0.3345	0.031	1.94E-07
		rs2229742	C	G	0.241	0.1323	0.0501	1.49E-06
		rs2647254	A	G	-0.1436	0.3504	0.0312	4.03E-06
		rs34970912	C	G	-0.5213	0.9648	0.0939	2.88E-08
		rs4383328	A	T	-0.1889	0.2848	0.0329	9.57E-09
		rs4679900	T	C	0.1538	0.5494	0.0304	4.31E-07
		rs4770473	T	C	0.159	0.4888	0.0306	2.00E-07
		rs4838595	T	C	-0.2576	0.1217	0.0453	1.29E-08
		rs6054829	A	G	-0.276	0.9221	0.0601	4.43E-06
		rs62385557	T	C	0.279	0.0718	0.0597	2.94E-06
		rs6440670	A	G	-0.1915	0.7966	0.0367	1.79E-07
		rs7017774	T	C	0.146	0.506	0.0303	1.44E-06
		rs7705526	A	C	0.1811	0.3428	0.0338	8.52E-08
		rs7965945	T	C	-0.2095	0.8457	0.0424	7.92E-07
		rs8005306	A	C	0.322	0.0539	0.0684	2.51E-06
		rs80195828	T	C	0.2695	0.9189	0.0562	1.59E-06
		rs878175	T	C	-0.1903	0.8105	0.0385	7.49E-07
		rs9813840	T	C	0.4373	0.96	0.0859	3.58E-07
Hannum	mRS0-2vs3-6 adjusted severity	rs1005277	A	C	0.3006	0.2982	0.033	8.92E-20
		rs10786282	A	G	-0.36	0.2124	0.0362	2.61E-23
		rs11190130	T	C	0.147	0.3869	0.0305	1.44E-06
		rs114606172	T	C	0.3554	0.0538	0.0677	1.54E-07
		rs1200332	T	C	0.179	0.2061	0.0371	1.44E-06
		rs12196813	T	C	0.4614	0.0385	0.0963	1.64E-06
		rs12271802	A	G	0.364	0.0552	0.0765	1.95E-06
		rs12417758	T	C	-0.2093	0.5461	0.0304	6.22E-12
		rs139107270	A	T	0.4628	0.0376	0.0959	1.39E-06
		rs148436209	T	C	0.49	0.0334	0.0952	2.64E-07
		rs1598856	A	G	0.1858	0.4492	0.0296	3.63E-10
		rs16958455	T	C	-0.1533	0.6322	0.0314	1.09E-06
		rs1824965	T	C	-0.1456	0.4524	0.0308	2.24E-06
		rs1849209	T	G	-0.1647	0.7591	0.0347	2.07E-06
		rs1984968	A	G	0.2047	0.8451	0.0414	7.44E-07
		rs2014202	A	G	0.1696	0.3246	0.0315	7.23E-08
		rs2195114	T	C	-0.1614	0.3345	0.031	1.94E-07
		rs2229742	C	G	0.241	0.1323	0.0501	1.49E-06
		rs2647254	A	G	-0.1436	0.3504	0.0312	4.03E-06
		rs34970912	C	G	-0.5213	0.9648	0.0939	2.88E-08
		rs4383328	A	T	-0.1889	0.2848	0.0329	9.57E-09
		rs4679900	T	C	0.1538	0.5494	0.0304	4.31E-07
		rs4770473	T	C	0.159	0.4888	0.0306	2.00E-07
		rs4838595	T	C	-0.2576	0.1217	0.0453	1.29E-08
		rs6054829	A	G	-0.276	0.9221	0.0601	4.43E-06
		rs62385557	T	C	0.279	0.0718	0.0597	2.94E-06
		rs6440670	A	G	-0.1915	0.7966	0.0367	1.79E-07
		rs7017774	T	C	0.146	0.506	0.0303	1.44E-06
		rs7705526	A	C	0.1811	0.3428	0.0338	8.52E-08
		rs7965945	T	C	-0.2095	0.8457	0.0424	7.92E-07
		rs8005306	A	C	0.322	0.0539	0.0684	2.51E-06
		rs80195828	T	C	0.2695	0.9189	0.0562	1.59E-06
		rs878175	T	C	-0.1903	0.8105	0.0385	7.49E-07
		rs9813840	T	C	0.4373	0.96	0.0859	3.58E-07
Hannum	ordinalmRS	rs1005277	A	C	0.3006	0.2982	0.033	8.92E-20
		rs10786282	A	G	-0.36	0.2124	0.0362	2.61E-23
		rs11190130	T	C	0.147	0.3869	0.0305	1.44E-06
		rs1200332	T	C	0.179	0.2061	0.0371	1.44E-06
		rs12196813	T	C	0.4614	0.0385	0.0963	1.64E-06
		rs12271802	A	G	0.364	0.0552	0.0765	1.95E-06
		rs12417758	T	C	-0.2093	0.5461	0.0304	6.22E-12
		rs139107270	A	T	0.4628	0.0376	0.0959	1.39E-06
		rs148436209	T	C	0.49	0.0334	0.0952	2.64E-07
		rs1824965	T	C	-0.1456	0.4524	0.0308	2.24E-06
		rs1849209	T	G	-0.1647	0.7591	0.0347	2.07E-06
		rs1984968	A	G	0.2047	0.8451	0.0414	7.44E-07
		rs2014202	A	G	0.1696	0.3246	0.0315	7.23E-08
		rs2195114	T	C	-0.1614	0.3345	0.031	1.94E-07
		rs2229742	C	G	0.241	0.1323	0.0501	1.49E-06
		rs2647254	A	G	-0.1436	0.3504	0.0312	4.03E-06
		rs4383328	A	T	-0.1889	0.2848	0.0329	9.57E-09
		rs4679900	T	C	0.1538	0.5494	0.0304	4.31E-07
		rs4770473	T	C	0.159	0.4888	0.0306	2.00E-07
		rs4838595	T	C	-0.2576	0.1217	0.0453	1.29E-08
		rs6054829	A	G	-0.276	0.9221	0.0601	4.43E-06
		rs62385557	T	C	0.279	0.0718	0.0597	2.94E-06
		rs6440670	A	G	-0.1915	0.7966	0.0367	1.79E-07
		rs7017774	T	C	0.146	0.506	0.0303	1.44E-06
		rs7705526	A	C	0.1811	0.3428	0.0338	8.52E-08
		rs7965945	T	C	-0.2095	0.8457	0.0424	7.92E-07
		rs8005306	A	C	0.322	0.0539	0.0684	2.51E-06
		rs9813840	T	C	0.4373	0.96	0.0859	3.58E-07
Hannum	ordinalmRS adjusted severity	rs1005277	A	C	0.3006	0.2982	0.033	8.92E-20
		rs10786282	A	G	-0.36	0.2124	0.0362	2.61E-23
		rs11190130	T	C	0.147	0.3869	0.0305	1.44E-06
		rs1200332	T	C	0.179	0.2061	0.0371	1.44E-06
		rs12196813	T	C	0.4614	0.0385	0.0963	1.64E-06
		rs12271802	A	G	0.364	0.0552	0.0765	1.95E-06
		rs12417758	T	C	-0.2093	0.5461	0.0304	6.22E-12
		rs139107270	A	T	0.4628	0.0376	0.0959	1.39E-06
		rs148436209	T	C	0.49	0.0334	0.0952	2.64E-07
		rs1598856	A	G	0.1858	0.4492	0.0296	3.63E-10
		rs1824965	T	C	-0.1456	0.4524	0.0308	2.24E-06
		rs1849209	T	G	-0.1647	0.7591	0.0347	2.07E-06
		rs1984968	A	G	0.2047	0.8451	0.0414	7.44E-07
		rs2014202	A	G	0.1696	0.3246	0.0315	7.23E-08
		rs2195114	T	C	-0.1614	0.3345	0.031	1.94E-07
		rs2229742	C	G	0.241	0.1323	0.0501	1.49E-06
		rs2647254	A	G	-0.1436	0.3504	0.0312	4.03E-06
		rs34970912	C	G	-0.5213	0.9648	0.0939	2.88E-08
		rs4679900	T	C	0.1538	0.5494	0.0304	4.31E-07
		rs4770473	T	C	0.159	0.4888	0.0306	2.00E-07
		rs4838595	T	C	-0.2576	0.1217	0.0453	1.29E-08
		rs6054829	A	G	-0.276	0.9221	0.0601	4.43E-06
		rs62385557	T	C	0.279	0.0718	0.0597	2.94E-06
		rs6440670	A	G	-0.1915	0.7966	0.0367	1.79E-07
		rs7017774	T	C	0.146	0.506	0.0303	1.44E-06
		rs7705526	A	C	0.1811	0.3428	0.0338	8.52E-08
		rs7965945	T	C	-0.2095	0.8457	0.0424	7.92E-07
		rs8005306	A	C	0.322	0.0539	0.0684	2.51E-06
		rs80195828	T	C	0.2695	0.9189	0.0562	1.59E-06
		rs9813840	T	C	0.4373	0.96	0.0859	3.58E-07
GrimAge	mRS0-1vs2-6	rs12116715	T	C	-0.266	0.0976	0.0557	1.76E-06
		rs13412647	A	G	0.3915	0.9625	0.0831	2.48E-06
		rs16936235	T	C	0.1908	0.831	0.0399	1.71E-06
		rs17094148	A	G	-0.18	0.7067	0.0323	2.55E-08
		rs17279885	A	G	-0.1737	0.2408	0.0348	5.87E-07
		rs1800440	T	C	-0.1834	0.8115	0.037	6.97E-07
		rs1837188	T	C	-0.1641	0.6459	0.032	2.96E-07
		rs1949808	C	G	0.1657	0.7035	0.0353	2.75E-06
		rs2010054	A	G	-0.1456	0.3972	0.0309	2.51E-06
		rs2813943	C	G	-0.154	0.631	0.0327	2.48E-06
		rs3817367	T	G	0.2347	0.8979	0.0498	2.46E-06
		rs4065321	T	C	-0.1703	0.5333	0.0296	8.78E-09
		rs6709296	T	G	0.151	0.4237	0.0302	5.57E-07
		rs6886532	T	C	-0.3752	0.0537	0.0806	3.19E-06
		rs72659772	T	C	0.2962	0.0708	0.0631	2.68E-06
		rs76183373	T	C	0.2802	0.0741	0.0598	2.78E-06
		rs773853	A	G	0.174	0.3486	0.0359	1.22E-06
		rs77488658	T	C	-0.2283	0.8912	0.0497	4.46E-06
		rs790051	A	G	-0.1676	0.2169	0.0355	2.34E-06
		rs7919238	T	C	-0.1787	0.7983	0.0386	3.75E-06
		rs9386796	T	C	0.1983	0.4597	0.0294	1.64E-11
		rs9790675	A	G	0.2246	0.8353	0.0418	7.52E-08
GrimAge	mRS0-1vs2-6 adjusted severity	rs12116715	T	C	-0.266	0.0976	0.0557	1.76E-06
		rs13412647	A	G	0.3915	0.9625	0.0831	2.48E-06
		rs16936235	T	C	0.1908	0.831	0.0399	1.71E-06
		rs17094148	A	G	-0.18	0.7067	0.0323	2.55E-08
		rs17279885	A	G	-0.1737	0.2408	0.0348	5.87E-07
		rs1800440	T	C	-0.1834	0.8115	0.037	6.97E-07
		rs1837188	T	C	-0.1641	0.6459	0.032	2.96E-07
		rs1949808	C	G	0.1657	0.7035	0.0353	2.75E-06
		rs2010054	A	G	-0.1456	0.3972	0.0309	2.51E-06
		rs2813943	C	G	-0.154	0.631	0.0327	2.48E-06
		rs3817367	T	G	0.2347	0.8979	0.0498	2.46E-06
		rs4065321	T	C	-0.1703	0.5333	0.0296	8.78E-09
		rs6709296	T	G	0.151	0.4237	0.0302	5.57E-07
		rs6886532	T	C	-0.3752	0.0537	0.0806	3.19E-06
		rs72659772	T	C	0.2962	0.0708	0.0631	2.68E-06
		rs76183373	T	C	0.2802	0.0741	0.0598	2.78E-06
		rs773853	A	G	0.174	0.3486	0.0359	1.22E-06
		rs77488658	T	C	-0.2283	0.8912	0.0497	4.46E-06
		rs790051	A	G	-0.1676	0.2169	0.0355	2.34E-06
		rs7919238	T	C	-0.1787	0.7983	0.0386	3.75E-06
		rs9386796	T	C	0.1983	0.4597	0.0294	1.64E-11
		rs9790675	A	G	0.2246	0.8353	0.0418	7.52E-08
GrimAge	mRS0-2vs3-6	rs12116715	T	C	-0.266	0.0976	0.0557	1.76E-06
		rs13412647	A	G	0.3915	0.9625	0.0831	2.48E-06
		rs16936235	T	C	0.1908	0.831	0.0399	1.71E-06
		rs17094148	A	G	-0.18	0.7067	0.0323	2.55E-08
		rs17279885	A	G	-0.1737	0.2408	0.0348	5.87E-07
		rs1800440	T	C	-0.1834	0.8115	0.037	6.97E-07
		rs1837188	T	C	-0.1641	0.6459	0.032	2.96E-07
		rs1949808	C	G	0.1657	0.7035	0.0353	2.75E-06
		rs2010054	A	G	-0.1456	0.3972	0.0309	2.51E-06
		rs2813943	C	G	-0.154	0.631	0.0327	2.48E-06
		rs3817367	T	G	0.2347	0.8979	0.0498	2.46E-06
		rs4065321	T	C	-0.1703	0.5333	0.0296	8.78E-09
		rs6709296	T	G	0.151	0.4237	0.0302	5.57E-07
		rs6886532	T	C	-0.3752	0.0537	0.0806	3.19E-06
		rs72659772	T	C	0.2962	0.0708	0.0631	2.68E-06
		rs76183373	T	C	0.2802	0.0741	0.0598	2.78E-06
		rs773853	A	G	0.174	0.3486	0.0359	1.22E-06
		rs77488658	T	C	-0.2283	0.8912	0.0497	4.46E-06
		rs790051	A	G	-0.1676	0.2169	0.0355	2.34E-06
		rs7919238	T	C	-0.1787	0.7983	0.0386	3.75E-06
		rs9386796	T	C	0.1983	0.4597	0.0294	1.64E-11
		rs9790675	A	G	0.2246	0.8353	0.0418	7.52E-08
GrimAge	mRS0-2vs3-6 adjusted severity	rs12116715	T	C	-0.266	0.0976	0.0557	1.76E-06
		rs13412647	A	G	0.3915	0.9625	0.0831	2.48E-06
		rs16936235	T	C	0.1908	0.831	0.0399	1.71E-06
		rs17094148	A	G	-0.18	0.7067	0.0323	2.55E-08
		rs17279885	A	G	-0.1737	0.2408	0.0348	5.87E-07
		rs1800440	T	C	-0.1834	0.8115	0.037	6.97E-07
		rs1837188	T	C	-0.1641	0.6459	0.032	2.96E-07
		rs1949808	C	G	0.1657	0.7035	0.0353	2.75E-06
		rs2010054	A	G	-0.1456	0.3972	0.0309	2.51E-06
		rs2813943	C	G	-0.154	0.631	0.0327	2.48E-06
		rs3817367	T	G	0.2347	0.8979	0.0498	2.46E-06
		rs4065321	T	C	-0.1703	0.5333	0.0296	8.78E-09
		rs6709296	T	G	0.151	0.4237	0.0302	5.57E-07
		rs6886532	T	C	-0.3752	0.0537	0.0806	3.19E-06
		rs72659772	T	C	0.2962	0.0708	0.0631	2.68E-06
		rs76183373	T	C	0.2802	0.0741	0.0598	2.78E-06
		rs773853	A	G	0.174	0.3486	0.0359	1.22E-06
		rs77488658	T	C	-0.2283	0.8912	0.0497	4.46E-06
		rs790051	A	G	-0.1676	0.2169	0.0355	2.34E-06
		rs7919238	T	C	-0.1787	0.7983	0.0386	3.75E-06
		rs9386796	T	C	0.1983	0.4597	0.0294	1.64E-11
		rs9790675	A	G	0.2246	0.8353	0.0418	7.52E-08
GrimAge	ordinalmRS	rs12116715	T	C	-0.266	0.0976	0.0557	1.76E-06
		rs13412647	A	G	0.3915	0.9625	0.0831	2.48E-06
		rs16936235	T	C	0.1908	0.831	0.0399	1.71E-06
		rs17094148	A	G	-0.18	0.7067	0.0323	2.55E-08
		rs17279885	A	G	-0.1737	0.2408	0.0348	5.87E-07
		rs1800440	T	C	-0.1834	0.8115	0.037	6.97E-07
		rs1837188	T	C	-0.1641	0.6459	0.032	2.96E-07
		rs1949808	C	G	0.1657	0.7035	0.0353	2.75E-06
		rs2010054	A	G	-0.1456	0.3972	0.0309	2.51E-06
		rs2813943	C	G	-0.154	0.631	0.0327	2.48E-06
		rs3817367	T	G	0.2347	0.8979	0.0498	2.46E-06
		rs4065321	T	C	-0.1703	0.5333	0.0296	8.78E-09
		rs6709296	T	G	0.151	0.4237	0.0302	5.57E-07
		rs6886532	T	C	-0.3752	0.0537	0.0806	3.19E-06
		rs72659772	T	C	0.2962	0.0708	0.0631	2.68E-06
		rs76183373	T	C	0.2802	0.0741	0.0598	2.78E-06
		rs773853	A	G	0.174	0.3486	0.0359	1.22E-06
		rs77488658	T	C	-0.2283	0.8912	0.0497	4.46E-06
		rs790051	A	G	-0.1676	0.2169	0.0355	2.34E-06
		rs7919238	T	C	-0.1787	0.7983	0.0386	3.75E-06
		rs9386796	T	C	0.1983	0.4597	0.0294	1.64E-11
		rs9790675	A	G	0.2246	0.8353	0.0418	7.52E-08
GrimAge	ordinalmRS adjusted severity	rs12116715	T	C	-0.266	0.0976	0.0557	1.76E-06
		rs13412647	A	G	0.3915	0.9625	0.0831	2.48E-06
		rs16936235	T	C	0.1908	0.831	0.0399	1.71E-06
		rs17094148	A	G	-0.18	0.7067	0.0323	2.55E-08
		rs17279885	A	G	-0.1737	0.2408	0.0348	5.87E-07
		rs1800440	T	C	-0.1834	0.8115	0.037	6.97E-07
		rs1837188	T	C	-0.1641	0.6459	0.032	2.96E-07
		rs1949808	C	G	0.1657	0.7035	0.0353	2.75E-06
		rs2010054	A	G	-0.1456	0.3972	0.0309	2.51E-06
		rs2813943	C	G	-0.154	0.631	0.0327	2.48E-06
		rs3817367	T	G	0.2347	0.8979	0.0498	2.46E-06
		rs4065321	T	C	-0.1703	0.5333	0.0296	8.78E-09
		rs6709296	T	G	0.151	0.4237	0.0302	5.57E-07
